# KSHV Reactivation and Novel Implications of Protein Isomerization on Lytic Switch Control

**DOI:** 10.3390/v7010072

**Published:** 2015-01-12

**Authors:** Jonathan Guito, David M. Lukac

**Affiliations:** Department of Microbiology, Biochemistry and Molecular Genetics, New Jersey Medical School and Graduate School of Biomedical Sciences, Rutgers Biomedical and Health Sciences, Rutgers University, Newark, NJ 07103, USA; E-Mail: ypw1@cdc.gov

**Keywords:** Kaposi’s sarcoma-associated Herpesvirus, Human herpesvirus-8, herpesvirus, reactivation, Rta, transcriptional activation, Pin1, Peptidyl-prolyl *cis*/*trans* isomerase

## Abstract

In Kaposi’s sarcoma-associated herpesvirus (KSHV) oncogenesis, both latency and reactivation are hypothesized to potentiate tumor growth. The KSHV Rta protein is the lytic switch for reactivation. Rta transactivates essential genes via interactions with cofactors such as the cellular RBP-Jk and Oct-1 proteins, and the viral Mta protein. Given that robust viral reactivation would facilitate antiviral responses and culminate in host cell lysis, regulation of Rta’s expression and function is a major determinant of the latent-lytic balance and the fate of infected cells. Our lab recently showed that Rta transactivation requires the cellular peptidyl-prolyl *cis*/*trans* isomerase Pin1. Our data suggest that proline‑directed phosphorylation regulates Rta by licensing binding to Pin1. Despite Pin1’s ability to stimulate Rta transactivation, unchecked Pin1 activity inhibited virus production. Dysregulation of Pin1 is implicated in human cancers, and KSHV is the latest virus known to co-opt Pin1 function. We propose that Pin1 is a molecular timer that can regulate the balance between viral lytic gene expression and host cell lysis. Intriguing scenarios for Pin1’s underlying activities, and the potential broader significance for isomerization of Rta and reactivation, are highlighted.

## 1. Kaposi’s Sarcoma-Associated Herpesvirus Latency and Reactivation: A Primer

Kaposi’s sarcoma-associated herpesvirus (KSHV), also known as human herpesvirus 8 (HHV-8), is a large double-stranded (ds) DNA virus [1,2,3,4,5]. KSHV causes Kaposi’s sarcoma (KS), an AIDS‑defining malignancy, and primary effusion lymphoma (PEL). Despite its discovery twenty years ago, it remains the most recently-identified human herpesvirus. KSHV is a *Rhadinovirus*, or γ2‑herpesvirus, classified together with MHV-68, HVS and rhesus rhadinovirus (RRV) [2,6]. KSHV diverged from *Lymphocryptovirus* or γ1-herpesviruses, such as Epstein-Barr Virus (EBV), *circa* 100,000 years ago in Africa [4]. The KSHV virion is enveloped and glycoprotein-studded, with large, ~120 nm icosahedral capsids [2,3,4,7,8]. Inside the envelope lies the tegument, an amorphous structure comprised of a multitude of viral and host proteins, although the functions of many remain unknown [2,3,4,9]. Inside the tegument lies the capsid that contains the embedded, linear viral genome [3,4]. 

The HHV-8 genome is variable in length, usually reported as between 160–170 kb [1,2,3,4,5,8]. Of this, 145 kb comprises unique sequence, while the remaining variable portion is derived of guanine-cytosine (GC)-rich terminal repeats (TRs) that flank the genomic ends [1,2,4,10]. Genomes contain ~87 open reading frames (ORFs) capable of encoding well over 100 functional gene products, a set of 15 KSHV‑unique “K” genes, up to ~25 unique viral microRNA (miRs) and a highly expressed noncoding transcript (nut-1, also known as polyadenylated nuclear RNA [PAN]) [1,2,3,4,5,10]. A large number of viral proteins are also involved in pathogenic functions within host cells, including for cell proliferation, paracrine signaling, immune suppression and inhibition of apoptosis [2,3,5].

Like all herpesviruses, KSHV can undergo two alternative, essential gene expression programs throughout its lifecycle: latency and lytic replication [2,3,4]. In nearly all infected cells, latency, defined by the absence of mature virus production, predominates within 24–48 h after initial infection [2,3,4]. Once adopted, the nonproductive latency program is characterized by constitutive expression of a small subset of KSHV genes, many of which are localized to a single locus [2,4,10]. The program is well documented to occur in both virus-harboring KS spindle cells and PEL cells [2,3,4,10,11,12,13,14].

While latency is the default state of KSHV, a small subpopulation of infected cells, usually 1% to 5%, support spontaneous lytic reactivation [2,3,4,7,10,14,15]. The lytic cycle is essential for production of progeny virus that can then disseminate and infect other cells and other individuals through shedding [2,3,4,7,10,14,15]. While virion production is the ultimate step in reactivation, it is by no means the predestined outcome. Sometimes, lytic reentry is abortive, or “sublytic,” and does not proceed to virion assembly and release [1,2,4,7,16]. This is because the herpesviral lytic cycle is regulated at several stages. Lytic reactivation can be thought of as a multistep cascade consisting of five broad kinetic intervals: immediate-early (IE) viral gene expression; delayed-early (DE) gene expression; viral DNA replication; late gene (L) expression; and finally virion production [2,3,4,7,10,14,17]. 

IE genes express a few viral transcription factors such as the lytic switch Rta (ORF50), which then activate the expression of DE genes, many of which are lytic cycle-specific K genes [2,4,7,17,18]. Notably, KSHV is unique among human herpesviruses in that many of these lytic K proteins, and other DE proteins, are mimics of cellular proto-oncoproteins and cytokines [2,4,5,9,15,17,18]. After initiation of viral DNA replication, late gene production begins [2,3,4,7,14,17]. These mostly comprise the aforementioned capsid, tegument and envelope proteins required for virion assembly [2,4,9]. It is not currently understood how late gene synthesis is regulated by viral DNA replication itself, independently of DE gene expression. Replication-dependent epigenetic regulation, such as histone modification, is among the possibilities [7,19,20,21]. Envelopment leads to maturation of virions, complete with decoration of envelopes with viral glycoproteins, such as K8.1 [3,4,22,23]. It is currently thought that infected B cells, the viral reservoir, release virions that can then disseminate to the lymphatic endothelium and seed for KS tumor development [2,4].

Lytic reactivation has been widely accepted in the literature as not just important for dissemination of infectious virus, but also as fundamental to tumorigenesis directly, a contention that is supported by animal models [2,3,4,7,8,10,11,13,15,23,24,25,26,27,28,29]. The virus may complement its latent tumorigenic potential by expression of the DE oncoproteins, some of which have transforming properties alone *in vitro* and in infected cells [2,24,30,31,32]. It is hypothesized that the secretion of paracrine factors, such as cytokines and growth factors, during the lytic cycle serves to stimulate the surrounding tumor microenvironment of uninfected and latently-infected cells for further growth and survival [2,4,10,11,15,33].

## 2. Function and Regulation of Rta Lytic Switch Protein

Replication and transcription activator (Rta) is a 691 amino acid (aa) IE transcription factor encoded from the major IE locus tricistronic transcript [2,34,35]. Its transcript is among the first produced following chemical induction by 12-*O*-tetradecanoyl phorbol-13-acetate (TPA), being expressed within 1 hpi [2,17,34]. This gene alone was identified to encode the lytic switch protein of KSHV, and is necessary and sufficient for the onset of productive lytic reactivation, with concomitant release of infectious virions [2,4,7,34,35]. Rta’s function was confirmed via observations that ectopic Rta induced reactivation alone in infected B cells, as well as by functional binding analyses with truncation and dominant negative (DN) mutants and by genetic analyses with Rta-deficient viral bacmid-infected cells, both of which were incapable of reactivation [2,7,35,36,37]. Further, addition of TPA could not induce the Rta-deficient virus, but induction was rescued by ectopic Rta expression [36]. Rta also autoactivates its own promoter, an activity characteristic of protein switches [7,37,38,39]. 

Both Rta and basic leucine zipper K-bZIP (K8) are syntenic orthologs of EBV transcriptional transactivators Rta (BRLF1, 20%) and Zta (BZLF1, 22%), respectively [7,34,37,40]. While KSHV Rta is alone required for viral reactivation through its transactivation activity at downstream viral promoters, both EBV transactivators are necessary for the EBV lytic program, in which they function independently and synergistically at different subsets of viral promoters [7,37,40,41]. K-bZIP, meanwhile, despite a homology to Zta and its role in gene expression, could not reactivate KSHV or transactivate viral genes alone [4,7,37,41]. 

**Figure 1:**
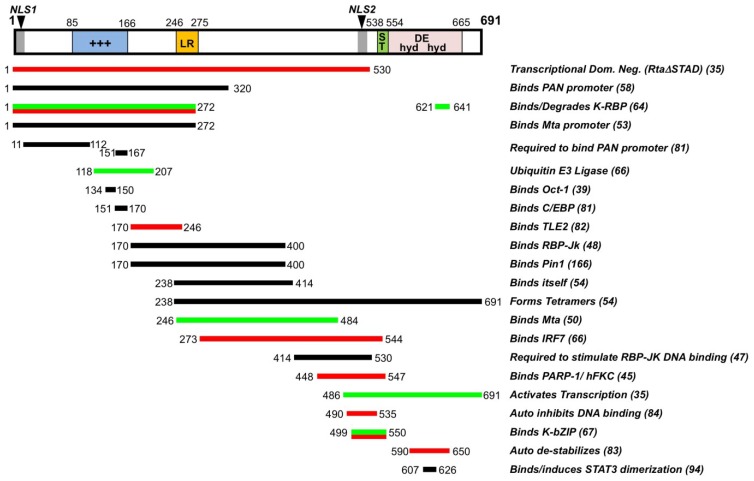
Rta/ORF50 primary amino acid structure/function map. A linear representation of the primary structure and predicted regions and interactions of Rta protein. Numbers refer to aa position. Locations of each domain are shown by the colored bars, with functional boundaries indicated by aa, corresponding to the activity or interacting protein listed in the column at right. Numbers in parentheses indicate references. Color codes for bars are: black, core functional domain; red, Rta inhibitor; green, Rta stimulator. Citations are listed in parentheses, and described in the text. +++, basic amino acid rich; LR, leucine heptapeptide repeat domain; ST, serine/threonine-rich; hyd/DE/hyd, repeats of hydrophobic and acidic amino acids, comprising Rta’s transactivation domain (TAD); NLS, nuclear localization sequence; Dom. Neg., dominant negative. Figure and legend modified from [37].

Rta protein has an apparent molecular weight of 73.7–120 kilodalton (kDa), a difference indicative of its extensive posttranslational modifications, predominantly phosphorylation (20 kDa alone) [7,34,37,42,43,44]. Rta is also ADP-ribosylated [45], and may contain other modifications. Rta encodes a multitude of structural and functional domains: an N-terminal DNA-binding domain (DBD), C‑terminal transactivation domain (TAD), basic amino acid-rich region, proline-rich regions, serine/threonine-rich region, cysteine/histidine-rich region, hydrophobic-acidic repeat region, leucine heptapeptide repeat domain, two nuclear localization signals (NLSs) and dimerization and tetramerization domains, in addition to a variety of sites and regions important for interactions with viral and cellular proteins (Figure 1) [2,4,7,37,40,46]. Removal of Rta’s TAD, which resembles a domain in viral protein (VP) 16 of herpes simplex virus 1 (HSV-1), results in a DN mutant (RtaΔSTAD) incapable of transactivation alone; this will be discussed further below [7,35,37,47]. 

### 2.1. Mechanisms of Rta-Mediated Transactivation

While Rta is both necessary and sufficient for viral reactivation, its transactivation activity is inefficient. This is underscored by reports demonstrating that Rta activity alone, in the absence of ongoing cellular or viral protein production, is unable to induce the full repertoire of lytic genes [7,16,37,46,48,49,50,51]. Rta’s inefficiency is also supported by data showing that Rta is weakly or nontransactive when mutated to prevent binding of specific cofactors, or when cofactor binding motifs in promoters are disrupted [7,16,37,39,46,47,49,52,53,54,55]. Finally, Rta expression does not necessarily translate into productive replication in infected cells, as typically fewer than 20% of Rta-positive PEL cells coexpress true late protein K8.1 (produced only following viral replication, and often utilized as a reactivation marker) [37,50]. The implications of these findings, are twofold. First, that Rta requires viral and cellular protein interactions to guide it through the full lytic cascade, via direct binding, posttranslational modifications or both. Second, that the conserved inefficiency of Rta lytic switch function may be important for KSHV pathogenesis. 

Rta’s transcription at downstream gene promoters is highly complex. In broad terms, and with exceptions, specificity to target promoters can be characterized as either direct or indirect, and as independent or dependent on interaction with the Notch signaling pathway effector recombination signal binding protein (RBP-Jk, which will be discussed below) [2,4,7,37,38,39,46,55,56]. Direct transactivation occurs by Rta binding to Rta-responsive elements (RREs) within certain promoters [4,37,38,46,55,56,57,58]. RREs, both RBP-Jk-dependent and -independent, can vary significantly, but four general consensus sequences have so far been uncovered: the palindromic repeat TTCCAGGAT(N)TTCCTGGGA, where N represents as many as sixteen random bases; multiple units of an A/T trinucleotide repeat, found in the K-bZIP, DE gene Mta and glycoprotein-encoding gene K1 promoters; recently-identified, TATA-box proximal elements known as “CANT DNA repeats” (discussed below); and the interferon (IFN) stimulated response element (ISRE)-like motif (A/G)NGAAANNGAAACT, found in promoters for vIL-6, vGPCR and ORF8 [37,38,46,55,56]. Binding to the latter depends on partial homology of Rta’s DBD to IRF family members [37,46]. Meanwhile, Rta binding affinity is largely proportional to the extent of transactivation for RBP-Jk-independent promoters [7,37]. The prototypical genes are kaposin and nut-1, with Rta binding at the nut-1 promoter with nanomolar (nM) affinity, and with nut-1 being the most abundant transcript produced during the lytic cycle [2,35,37,53,59,60]. Direct Rta transactivational targets have been identified by several labs. One such screen from our lab reported eight direct targets, including the promoters for nut-1, Mta, viral interleukin (vIL)-6, viral shutoff exonuclease (vSOX) and vOX2 [57]. Additional promoters, such as ORF45 and the miRNA locus, have also been described, though use of different methods and cell lines makes confirmation of authentic direct promoters difficult [37,38,56]. Finally, Rta also can bind DNA combinatorially with cellular or viral cofactors, such as octamer 1 (Oct1) and others, or enhance their transactivation, such as CREB binding protein (CBP); these are discussed below [4,37,39,46,52]. 

### 2.2. Rta Positively and Negatively Interacts with Host and Viral Cofactors

Rta is involved in a host of other important lytic cycle functions beyond its primary role as a transcription factor. For one, viral DNA replication is unable to proceed without Rta activity at oriLyts [7,37,61]. There, Rta binds to RREs as an origin binding protein in conjunction with K-bZIP and CCAAT/enhancer binding protein (C/EBP)-α (which itself has palindromic motifs within oriLyts) [37,55,61]. Together, these proteins recruit the core replication machinery to viral genomic DNA [7,61]. Rta also inhibits p53 transcription via a direct interaction with CBP, and modulates both IFN regulatory factor (IRF)7 and cellular K-Rta binding protein (K-RBP) stability via Rta’s E3 ubiquitin ligase (Cys/His-rich) domain [62,63,64,65,66]. Rta ubiquitylates the Notch and hypoxia response pathway target protein Hey1, a transcriptional repressor that may be involved in cell differentiation; it has been further suggested, but not confirmed, that Rta can destabilize latency-associated nuclear antigen (LANA)-1 and K-bZIP [7,46,64,67,68]. Rta’s ubiquitylation of some of the above proteins is in response to their own repressive function against Rta; in fact, multiple factors positively and negatively regulate Rta expression and activity for tight control over reactivation from latency. 

A number of antagonistic factors stem from viral latency itself. LANA, for instance, is capable of repressing Rta at multiple levels. LANA inactivates transcription from the ORF50 promoter directly, as well as indirectly, by competitive binding with cofactors CBP and RBP-Jk to prevent Rta-mediated autoactivation [4,7,14,46,69,70,71,72,73]. LANA also may recruit histone deacetylases (HDACs) and specificity protein 1 (Sp1) for similar repression from their motifs in the Rta promoter [2,4,14,20,52,71,73,74]. LANA acts directly on Rta protein to prevent Rta autoactivation [2,4,7,70]. Two miRs directly target the 3' untranslated region (UTR) of Rta mRNA for degradation [75,76,77,78]. vFLIP can repress Rta transactivation activity, as well as Rta’s own transcription through its effects on nuclear factor of kappa B (NF-κB) [7,79]. vFLIP mediates this repression in at least two ways. First, through NF-κB’s competition with, and sequestration of, RBP-Jk for DNA binding and for Rta protein association, respectively [7,14,80]. Second, through inhibition of the activating protein (AP)-1 pathway, blocking Rta expression [79,81]. As AP-1 binding sites are found in both the promoters of Rta and downstream genes as well as in oriLyts, repression of AP-1 transactivation likely affects both Rta expression and functions as a transcription factor and DNA replication regulator [7,37,61,81].

Cellular and viral lytic cycle proteins also modulate Rta [37,82,83,84]. In addition to HDAC and Sp1, K-RBP and IRF7 block Rta transactivation activity, the former through an additional interaction with cellular transcription intermediary factor 1β (TIF1β), which are thought to bind to specific promoter DNA elements and block Rta function (K-RBP) or compete for Rta DNA-binding (IRF7) [7,63,64,65]. Meanwhile, poly(ADP-ribose) polymerase 1 (PARP1) and human kinase from chicken (hKFC) bind directly to Rta protein’s Ser/Thr-rich domain to modify Rta through ribosylation and phosphorylation, respectively, inhibiting Rta transactivation [45]. Rta-modulating phosphorylation is also induced by the proliferation and apoptosis regulator Akt (also known as protein kinase B) of the PI3K pathway [7,85]. Finally, K-bZIP, a cofactor with Rta for initiation of viral replication at oriLyts, also inhibits Rta transactivation of selective viral promoters, including for nut-1, Mta and K-bZIP itself, by directly binding to Rta [2,4,7,37,67]. K-bZIP is also known to bind and repress CBP, which might disrupt Rta expression and function [4,7,67]. 

Thus, also, do multiple proteins, beyond K-bZIP in the context of DNA replication, enhance Rta’s expression and functions. For instance, Pim1 and Pim3, proto-oncoproteins involved in cell cycle and apoptotic pathways, upregulate Rta autoactivation by binding to and repressing LANA’s inhibition of the Rta promoter [86]. Viral G protein coupled receptor (vGPCR) may also negate LANA inhibition by reducing HDAC activity to allow for Sp1- and Sp3-dependent Rta promoter activity [87,88]. Hypoxia inducible factor (HIF)-1α can directly activate the Rta promoter, due to the presence of several putative hypoxic response elements (HREs) [69,89,90]. Interestingly, LANA was reported to activate Rta transcription through binding to HIF-1α at HREs during hypoxia, suggesting a context-dependent function for LANA and a mechanistic explanation for hypoxia-driven reactivation [69]. It remains unclear, however, how constitutively active HIF-1α is incapable of inducing Rta expression in the absence of hypoxia. X-box binding protein 1 (XBP-1), a critical inducer of the unfolded protein response (UPR) activated in stress conditions (including hypoxia) by the endoplasmic reticulum (ER) sensor BiP (Grp78), also directly transactivates Rta at ACGT-containing elements [91,92]. As XBP-1 activity induces B cell differentiation into secretory plasma-like cells, this process may be important for viral pathogenesis [91]. The aforementioned CBP, as well as p300, are transcriptional coactivators with intrinsic histone acetyltransferase (HAT) activity that bind to Rta at downstream Rta target promoters [7,37,52]. AP-1 is a complex of c-Jun and c-Fos proteins and may contribute the strongest transactivation activity for Rta expression, as evidenced by induction of a productive replication cycle by TPA that is similar to Rta-mediated induction [2,4,7,14,79,81,93]. Signal transducer and activator of transcription 3 (STAT3), a growth factor- and cytokine-responsive regulator, is dimerized by Rta, allowing STAT3 to translocate to the nucleus and induce STAT3 transcriptional targets [94]. Rta also binds to C/EBP-α, and recruits basal transcription complex Mediator and chromatin remodeling complex SWI/SNF, at Rta promoters to potentiate viral gene transcription [4,7,37]. Oct1 is a cofactor with Rta, with binding sites in the Rta promoter stimulating Rta autoactivation, and conversely, in the LANA promoter to upregulate LANA as a negative feedback circuit [4,7,37,39]. Oct1 is also necessary for transactivation of the K-bZIP promoter [37,39]. K-bZIP itself can work cooperatively with Rta to facilitate transcription at select promoters, including for Rta, vIL-6, Mta and K-bZIP; as K-bZIP seems to both activate and repress its own promoter and have dual functions with Rta, its regulation of lytic replication is presumably complex [7,37]. 

Finally, Mta, in addition to its functions described above, binds to and enhances Rta transactivation of selective downstream viral genes [37,50,55,95]. Loss or mutation of Mta showed that it is required for productive replication [37,50]. It is one of the first lytic genes expressed, one of the few directly targeted by Rta, and Mta protein can activate promoters in concert with Rta, including for itself, nut-1, Rta, kaposin and viral thymidine kinase (vTK) [37,50,81]. Mta also has transactivation potential alone in some contexts, as it can activate transcription of the nut-1 promoter independently of Rta; posttranscriptional roles likely exist for certain promoters as well, such as for nut-1 and viral DNA polymerase [37,50,95,96]. Mta binds mRNA and stabilizes a variety of transcripts, and one potential model is that Mta synergizes with Rta for transcriptional initiation and then enhances elongation by binding to and stabilizing nascent transcripts, where it may remain bound to enact its downstream activities [37,95,96]. Mta’s importance in productive replication was highlighted by data showing that, despite the aforementioned dearth of Rta-expressing cells positive for reactivation as indicated by K8.1 expression (fewer than 20%), more than 80% of Mta-expressing cells were reactivated [37,50]. As Mta represents a much better predictor of virus proceeding through a complete lytic cascade, it has been characterized as a “commitment factor” that drives inefficient Rta function in the direction of productive replication [37,50]. 

### 2.3. RBP-Jk Is Essential for Rta-Mediated Transactivation

Of all single Rta cofactors, however, canonical Notch pathway effector RBP-Jk (also known as CSL, for EBV core promoter-binding factor [CBF]-1/suppressor of hairless [Su(H)]/longevity assurance gene [Lag]-1) is the only one shown to be essential for Rta transactivation activity at viral and cellular promoters and for productive reactivation [4,7,37,47,48,49]. The Notch pathway is one of the oldest evolutionarily conserved signaling pathways in multicellular organisms [97]. It is involved primarily in development and cell fate, including intercellular communication and stem cell differentiation [97]. It also regulates apoptosis and angiogenesis, and Notch pathway dysregulation, which causes self-renewal and angiogenic tumor growth, is implicated in a variety of lymphoid cancers, such as T cell leukemias [97,98,99,100]. When signals including vascular endothelial growth factor (VEGF) and other cytokines induce Notch ligands Jagged or Delta-like to interact with one of the four human Notch single-pass transmembrane receptors, cleavage events release the Notch intracellular domain (NICD), which translocates to the nucleus and binds to RBP-Jk [46,97,98,100,101,102,103,104]. In the canonical Notch pathway, prior to NICD association, RBP-Jk is constitutively bound to promoter targets as part of HDAC corepressor complexes, at a (C/T)GTGGGAA consensus motif, and represses transcription [37,48,55,97]. NICD binding disrupts this repression, allowing it to recruit HAT proteins, signal through activated RBP-Jk and transcribe downstream Notch pathway genes, which include Hey and Hes family repressors [97,98,101,103,104]. 

In KSHV-infected cells induced for lytic reactivation, Rta associates with RBP-Jk in order to transactivate downstream viral and cellular genes [37,46,47,48,49]. Many of Rta’s gene promoter targets are RBP-Jk-dependent (though many also require, or are enhanced by, additional interacting proteins, some of which were described above), including Mta, K-bZIP, LANA, vGPCR, IL-6, Hes1, vTK, modulator of immune recognition (MIR)1 and MIR2, vCCL1 and others [4,16,37,38,46]. In fact, RBP‑Jk binding has been identified to at least 99 sites within the KSHV genome in infected cells ( [105], and as many as 34 Rta transcriptionally-activated viral genes have been described; this suggests the potential for an Rta-RBP-Jk complex to induce the entire lytic cascade [37,106]. 

Proof for RBP-Jk as a cofactor in Rta-mediated transactivation required for productive replication came in the form of truncation and mutation analyses of both proteins as well as target promoters [7,16,36,37,47,48,49,51,54]. The prototypical promoter for characterization of RBP-Jk interactions is Mta. RBP-Jk binding sites lie proximal to Rta binding elements [37,47,55]. Alterations to either of these sites reduced or prohibited Rta and/or RBP-Jk binding, transactivation or both, depending on location of a mutation within the promoter or on rearrangement between particular elements [37,47,55]. Independent binding of each protein at promoters, and subsequent ternary complex formation with promoter DNA, was required for optimal transactivation [37,47,53,55]. While RBP-Jk DNA binding was necessary for transactivation of Mta, RBP-Jk was found, unusually, not to constitutively bind to KSHV promoters in the absence of Rta, in sharp contrast to its mechanism for canonical Notch signaling [7,37,47,48,49]. This was determined by lack of RBP-Jk enrichment on viral promoters during latency, and by evidence that a constitutively active RBP-Jk mutant fused to the TAD of HSV-1 protein VP16 (RBP-Jk/VP16) was unable to bind to promoters alone [37,47,49]. Rta DNA binding, meanwhile, was determined to not be sufficient for transactivation at some RBP-Jk-dependent promoters [37,47,51]. An Rta mutant lacking its TAD, which begins at aa 530, but with its DBD (aa 1-272) intact (RtaΔSTAD), was also unable to activate its downstream genes alone; however, when combined with RBP-Jk/VP16, RtaΔSTAD rescued RBP-Jk DNA-binding at the Mta promoter [37,47]. The interaction also rescued transactivation [37,47]. Meanwhile, RBP-Jk-null fibroblasts were deficient in transactivation at Mta, but not nut-1, which is a direct Rta target; ectopic expression of RBP-Jk rescued this activity [37,48,49]. Taken together, Rta binding to RBP-Jk appears to stimulate RBP-Jk DNA binding at Rta downstream promoters containing both Rta- and RBP-Jk-specific elements, and in conjunction with additional cofactors at certain promoters, activates gene transcription. 

While this basic model for RBP-Jk-dependent, Rta-mediated transactivation addressed many of the questions surrounding regulation of KSHV gene expression, it was still not fully understood how Rta physically bound to its promoter elements in complex with RBP-Jk. Originally, 40 nt and 26 nt sequences containing identical, 16-nt palindromic RREs were defined within the Mta and K-bZIP promoters adjacent to an RBP-Jk binding site, but as flanking mutations in the Mta promoter, including in TATA-proximal sequences, revealed profound defects to transactivation without affecting RBP-Jk binding, the architecture required for Rta binding developed into a more complex picture [7,37,46,47,48,55]. First, it was noted that Rta elements were present upstream and downstream of the RBP-Jk binding site [7,37,46,47,48,55]. Second, Rta bound with high affinity to A/T trinucleotide repeat units within these elements, and the number and position of elements corresponded to the strength of Rta DNA binding [7,37,46,47,48,55]. Third, DNA footprinting mapped to four sites, distal and proximal to the RBP-Jk element, with the proximal sites flanking both sides of the element [37,55]. These four sites overlapped with A/T repeats. It was determined that the sites shared the consensus sequence ANTGTAACANT(A/T)(A/T)T, known as the “CANT DNA repeat” [37,55]. These units were repeated seven times in the four sites, two of which formed palindromes [37,55]. Further, it was shown that CANT repeats are present at a variety of Rta responsive promoters adjacent to RBP-Jk binding motifs (including at oriLyts), and represent a broadly-applicable RRE that defines Rta-RBP-Jk ternary complex formation and transcriptional mechanics [37,46,55]. Rta binds relatively weakly to single CANT DNA elements or palindromes, but binds with nM affinity to the full cohort of 7 CANT repeats in the Mta promoter.

Rta is not alone in its ability to mimic the NICD and use RBP-Jk for KSHV’s own pathogenesis. Epstein-Barr virus nuclear antigen (EBNA) 2 also binds to RBP-Jk to transactivate EBV downstream genes in a manner analogous to NICD [2,4,7,34,37,41,46,103,104]. However, EBNA2 utilizes RBP-Jk by different means. For instance, RBP-Jk is required for EBNA2-mediated establishment and maintenance of latency, and their interaction depends solely on NICD-like binding to RBP-Jk’s beta-trefoil domain (BTD), which blocks the activity of the larger RBP-Jk central repression domain (CRD) [37,47,49,107]. This interaction is defined by the conserved RBP-Jk binding peptide signature GPPWWPP, shared by both EBNA2 and NICD [37,47,49,107]. Finally, neither EBNA2 nor NICD can optimally transactivate KSHV genes with RBP-Jk alone, save a few exceptions, and cannot induce KSHV lytic reactivation [37,47,49,107]. Rta, conversely, requires RBP-Jk for lytic reactivation; can bind to RBP-Jk’s BTD, as well as N-terminal domains; can recruit RBP-Jk to EBV promoters and upregulate latent genes; and, importantly, does not contain the seven-nt consensus binding peptide for its interaction with RBP-Jk, instead relying on a currently unknown, noncanonical motif [4,34,37,41,46,47,48,49,107]. 

While the motif itself still needs to be elucidated, it is clear based on functional binding studies that Rta interaction with RBP-Jk occurs within a 117 aa region of Rta between aa 414 and 530, just N-terminal to the Ser/Thr-rich domain and inclusive of the NLS [37,47]. This was further determined by transactivation analysis, in addition to direct RBP-Jk binding studies, in which RtaΔSTAD was further truncated to Rta aa 414 [37,47]. This Rta mutant, unlike RtaΔSTAD, was unable to rescue transactivation of the Mta promoter with RBP-Jk/VP16, nor was it able to form ternary complexes with RBP-Jk and promoter DNA in supershift assays [37,47]. Thus, these data suggested that the minimal Rta region required for binding RBP-Jk was aa 414–530; this domain functions in concert with Rta’s DNA binding domain to stimulate RBP-Jk DNA binding to Rta responsive promoters. Nevertheless, the requirement for this domain is in contrast to an N-terminal region in Rta, between aa 170 and 400, which was shown to bind to RBP-Jk in solution, but was not sufficient alone for ternary complex formation and transactivation [37,47,48]. Taken together, the aa 414–530 region of Rta is required for both binding by RBP-Jk and stimulation of transactivation-competent ternary complex formation with promoter DNA. 

Finally, in a study from our lab that bridged Rta CANT repeat recognition with its physical interaction with RBP-Jk at downstream promoters, it was revealed that RtaΔSTAD inhibited Rta‑mediated transactivation and lytic replication, suggesting that RtaΔSTAD acted as a DN against WT Rta [35]. Thus Rta formation of mixed multimers was a required for its function. Further analysis showed that of all multimers, tetramers were sufficient to mediate the ability of Rta to transactivate genes [37,54]. Functional binding studies mapped the minimal tetramerization domain of Rta to aa 244 to 414 [37,54]. This region was notable for its inclusion of a 31 nt, N-terminal leucine heptapeptide repeat domain (LR) [37,54]. The KSHV Rta LR is similar to leucine zippers (LZs) in yeast, and shares a similar structure to Rta homologs in other primates, including three conserved leucines spaced at seven-residue intervals [37,54]. LZs are known to form alpha helix-based coiled coils and play a role in protein dimerization [37,54,108,109]. However, the KSHV Rta LR is also divergent from LZs in that it contains a high proline content; the LR overlaps with Rta’s proline-rich region [37,54,108,109]. The five prolines within the LR are conserved among *γ-herpesvirinae*, and had originally been predicted to prevent coiled coil formation typically important for canonical LZ oligomerization [37,40,54,108,109]. As the KSHV Rta LR was necessary for tetramer formation, it was hypothesized that the region might enable this function without a need for the hypothetical coiled coil structure [37,54]. To ensure coiled coils weren’t required for higher order Rta, the conserved leucines were mutated to prolines. The Rta-L3P mutant formed almost exclusively tetramers, confirming the nonessentiality for a typical LR structure in this activity [37,54]. Surprisingly, Rta-L3P was capable of WT levels of transactivation and reactivation [37,54]. It was concluded that Rta tetramers are essential for its transactivation potential and that, interestingly, the proline content within and beyond the LR, but not the LR’s canonical secondary structures, may be important in determining Rta’s higher order status—and perhaps broader, additional functions—based on their modification [37,54]. 

Given the body of evidence, a dynamic model for Rta transactivational function has been proposed: Rta protein forms tetramers and binds to RREs in viral and cellular promoters, alone or in conjunction with essential cofactors; straddling of Rta tetramers that contact multiple, flanking palindromic CANT DNA repeats, via binding of a novel Rta peptide motif to RBP-Jk, targets RBP-Jk to its element present in many Rta gene targets, allowing for the recruitment of additional coactivators and initiation of gene transcription [37,46,47,54,55]. Success of this transactivation program is critical to completion of the entire lytic cycle cascade, and relies on the interplay between Rta’s interaction with cofactors and, likely, on guidance by putative, proline-directed modifications that regulate Rta to carefully define its activities throughout viral reactivation [2,4,7,37,42,46,49]. It is the recent report published by our lab describing one such putative proline-directed modification of Rta—regulation by proline isomerization—that is the major focus of this review. 

## 3. Function, Regulation and Dysregulation of Pin1 Isomerase and its Novel Role in KSHV Lytic Reactivation

Posttranslational modifications are absolutely vital to the proper function of proteins within a cell, for signaling, conformation, interactions with other factors, stability, localization, DNA binding and transactivation, among many others. A number of potential modifications to Rta include phosphorylation, sumoylation, ubiquitylation and proline-directed modifications such as prolyl hydroxylation and prolyl isomerization [2,4,7,34,37,42,43,44,45,46,54,55,64,68,110]. The demonstrated importance of prolines within Rta may not have been limited to a role in tetramerization, but could have broader consequences on Rta function. 

Such modification is possible by peptidyl-prolyl *cis*/*trans* isomerases (PPIases) [111]. Isomerization of proline was first discovered as an important mechanism for proper protein function in the context of nascent protein folding [111,112,113,114,115]. The ability of primary amino acid structure to correctly fold into a functional conformation following ribosomal synthesis within the ER is largely dictated by the physical properties of the amino acids themselves. As *trans* form residues are solely synthesized by ribosomes, any protein that requires *cis* form residues would be unable to correctly fold and function. However, proline isomerization, by itself, is a rate-limiting process occurring at the multi-minute timescale [115,116,117]. A cell would be unable to survive if its protein contents took so long to mature. 

Peptidyl-prolyl *cis*/*trans* isomerases (PPIases) are highly conserved cellular catalysts that bind to and isomerize prolines at millisecond timescales, thus, effectively allowing for rapid, physiologically-relevant protein folding and function [111,113,115,116]. They are found in all organisms, including bacteria. There are four classes of PPIases: cyclophilins (Cyps), FK506-binding proteins (FKBPs), parvulins and the protein Ser/Thr phosphatase 2A (PP2A) activator (PTPA) [111,113,115,116]. The initial characterization of Cyps and FKBPs revealed them as targets for immunosuppressive and anticancer drugs cyclosporine A, FK506 and rapamycin, though it was soon reported that this was unrelated to their PPIase activity [111,113,114,116]. Further, their biological significances were questioned due to their redundancy, the presence of dedicated chaperone molecules and that disruption of single or multiple PPIase genes did not affect cell viability [111,114,118]. It did not appear that PPIases were essential general factors, although in subsequent years, important specific interactions were described. For FKBPs, for instance, FKBP12 was found to associate with ryanodine and inositol 1,4,5-triphosphate (IP_3_) receptor subunits and inhibit TGF-β receptors [111,113,114,115,119]. Interestingly, cyclophilins appear to be important in the pathogenesis of various virus, including HIV-1, hepatitis C virus (HCV) and human cytomegalovirus (HCMV, human herpesvirus 5) [111,113,114,115,120].

### 3.1. Human PPIase and Cell Cycle Regulator Pin1

**Figure 2:**
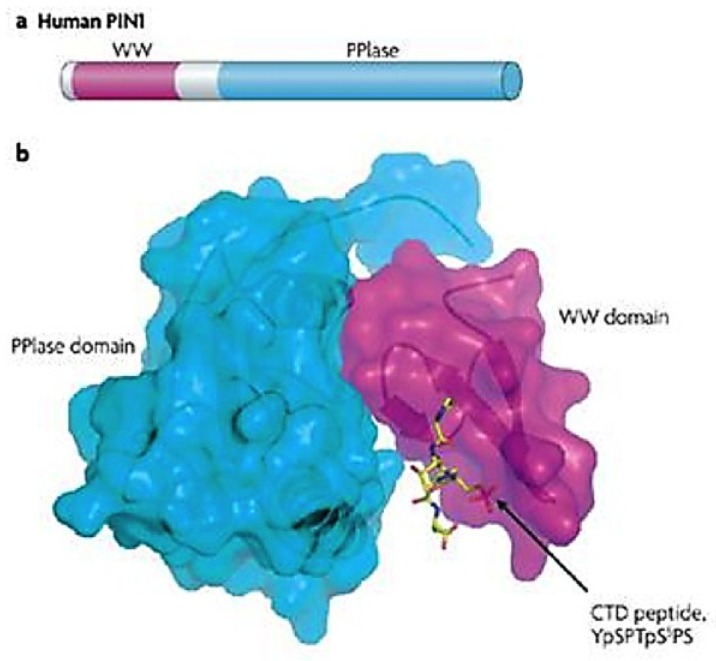
Pin1 prolyl isomerase protein structure. Secondary amino acid (**a**) andSpace-filling (**b**) models show Pin1, a small, ~18 kDa protein consisting of two domains: a WW binding domain (purple) named after two invariant tryptophans, and a peptidyl-prolyl isomerase (PPIase) domain (blue) that catalyses *cis*-to-*trans* isomerization. The WW recognition motif is visualized by the RNA polymerase (RNAP) II carboxyl terminal domain (CTD) peptide, which, unique to cellular isomerases, is a phosphorylated serine or threonine directly N-terminal to a proline (pS/T-P motif). This motif is also acted upon by the catalytic binding pocket of the PPIase domain. The two domains are connected via a flexible loop linker (at top) in the PPIase domain that allows for inter-domain coordination. Figure reproduced with permission from Lu and Zhou, Nature Reviews Molecular and Cellular Biology; published by Nature Publishing Group, 2007 [127].

In 1996, a new class of PPIases, parvulins, was identified as the result of a screen in *Aspergillus nidulans* for direct binding inhibitors of the essential mitotic kinase never in mitosis A (NIMA) [116,121,122]. The screen isolated three human proteins, one of which was peptidyl-prolyl isomerase NIMA interacting protein (Pin)1, a small 18 kDa protein determined to be a novel PPIase containing characteristic N-terminal WW substrate binding and C-terminal PPIase catalytic domains (Figure 2) [121,123,124,125]. Despite sharing the same basic domains with similar enzymatic activity, Pin1 was found to have a dramatically different structure from the other PPIase classes (which themselves are structurally distinct) [111,115,123,126]. 

**Figure 3:**
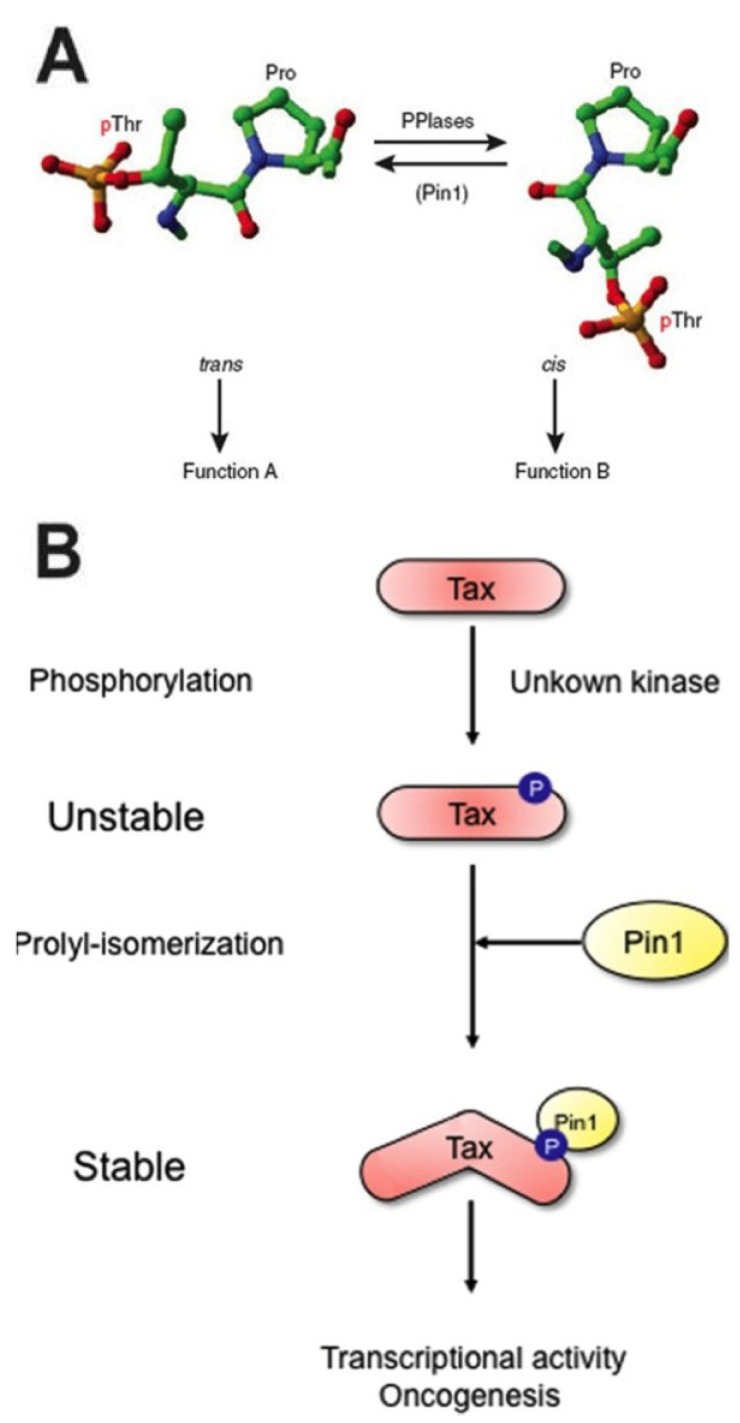
Mechanism of prolyl isomerization and its effect on substrate function. (**A**) *Cis*-*trans* isomerization is an intrinsically slow process; PPIase catalysis at pS/T-P motifs reduces this timescale from minutes to milliseconds, by binding to the phospho-residue N-terminal to proline, which the double-bonded oxygen and thus, the torsion barrier preventing conversion, allowing for a 180° rotation about the bond. (**B**) Since only *trans*-form of prolines bcan e acted on by regulators, isomerization can “lock” phosphorylation, and therefore a substrate’s function (such as, for example, stability of the human T cell leukemia virus (HTLV)-1 Tax oncoprotein, above), in place. As such, Pin1 is regarded as a timer of a variety of critical cell cycle and signaling events, including for those involving both cellular and viral regulators. Figure reproduced with permission from Lu *et al.*, Nature Chemical Biology; published by Nature Publishing Group, 2007 [115], and from [131].

Pin1 showed conservation from yeast (where it is known as Ess1) to humans [111,115,116,121,123,128]. Its role as a suppressor of NIMA-induced mitotic catastrophe marked the first non-“housekeeping” function ascribed to isomerases, in that it was both essential for cell viability in general, and as a regulator of mitosis specifically [121]. Additional characterization of Pin1 showed an intriguing specificity for peptidyl-prolyl motifs that required phosphorylation of the N-terminal peptidyl residue for recognition and for isomerization [115,116,123,129]. Peptidyl residues must be, in the case of Pin1, phosphoserines or phosphothreonines. It appeared that Pin1 WW domains bound to pS-P or pT-P (known as “pS/T-P motifs”) in targets by recognition of the phosphorylation, which reduced the double-bonded character of the oxygen N-terminal to the peptide bond (Figure 3A) [115,116,123,126,130]. As for all PPIases, this significantly reduced the torsion barrier that restricted rotation about the peptide bond from *trans* to *cis* forms (or *vice versa*), rapidly speeding up the conversation rate by several orders of magnitude [111,115,116,126].

The findings pertaining to Pin1’s phosphorylation dependency were significant for a few reasons. First, phosphorylation greatly slows the spontaneous *cis*-to-*trans* conversion rate and makes catalyzed, reversible isomerization essential for proteins requiring a particular conformation for function [115]. Second, no other PPIase recognized motifs that contained phosphorylated residues [111,115,123]. Third, and most importantly, action at phosphorylated moieties strongly implied that Pin1 has broadly-applicable regulatory potential at a previously unrecognized, postphosphorylational level [115,130,132,133]. This is because kinases and phosphatases are involved in numerous cell signaling events and are capable of targeting *trans* form serines and threonines only [115]. Thus, Pin1 binding to specific motifs within important regulatory or effector proteins render phosphorylation (or lack thereof) “locked in” by a switch to *cis* form, resistant to kinase or phosphatase activity. Protracted “on” or “off” states under the control of Pin1 isomerization, then, alter protein function and subsequently the conduct of their constituent pathways (Figure 3B).

### 3.2. Dysfunction of Pin1 Is Often Associated with Tumorigenesis

It was quickly borne out that Pin1 was indeed an integral cell-signaling regulator [115,121,123,130]. Perhaps its best-studied, and most important single interaction lies with Cyclin D and the G_1_/S checkpoint, a prime example of Pin1’s postphosphorylational control [115,119,130,133,134,135,136]. Pin1’s role, however, extends beyond cell cycle signaling; it is a truly pleiotropic enzyme with a wide array of substrates (Table 1).

Under normal conditions in noncancerous tumors, evidence suggests that Pin1 acts in a general tumor suppressive capacity [115,121,128,130,132,133,137,138,139,140,141]. Overexpression of Pin1, however, is attributed to a large number of malignancies at both the tumor and molecular levels [119,127,133,134,135,142,143,144,145,146,147,148]. Pin1 has been implicated in colorectal cancer (β-catenin), breast cancer (Cyclin D, AP-1, Akt, centrosome duplication, Notch1), prostate cancer (TRK-fused gene [TFG]), glioblastoma (NF-κB), hepatocellular carcinoma (HCC, p70S6K, β-catenin) and acute myeloid leukemia (AML, AP-1) [134,135,142,143,144,145,147,148,149,150,151,152]. The particular Pin1-dysregulated substrates and pathways are not mutually exclusive, and many of them are affected in cell type- and tumor type-specific combinations. Importantly, in a comprehensive study of over 2000 human tumors representing 60 types of cancer, Pin1 was found to be at least 10% overexpressed in 38 of the 60 tumor types, especially for breast, colon and prostate cancers [135]. In support of this, a clinical study of nearly 600 prostate cancer patients, Pin1 was strongly associated with cancer severity and recurrence risk [153]. 

Finally, Pin1 was also more recently implicated in virally-derived tumors as well as in viral pathogenesis in general. For instance, HIV-1 capsid (CA) protein uncoating, a process essential for subsequent reverse transcription and viral replication, is mediated by Pin1 activity [154,155]. So too does Pin1 interact with and inhibit APOBEC3G, a cytidine deaminase and antiviral factor that incorporates into HIV-1 virions to block viral replication, as well as stabilizes the HIV-1 integrase for incorporation of virus into host genomes [156]. Pin1 promotes ubiquitylation of IRF3, inhibiting the host IFN innate antiviral response and promoting susceptibility to viral infection [157]. For hepatitis C virus, Pin1 interacts with viral nonstructural proteins NS5A and NS5B to enhance HCV replication [158]. Hepatitis B virus (HBV) encoded protein X (HBx) stability is mediated by Pin1, which is associated with HCC [159]. Notably, Pin1 stabilizes the human T cell leukemia virus 1 (HTLV-1) oncoprotein Tax, a transcription factor similar to KSHV Rta, at least thematically, in that it transactivates downstream viral promoters for productive lytic replication and pathogenesis [131,133,160,161]. Stabilization allows Tax to interact with IKKγ and contribute to NF-κB-mediated cell transformation [161]. 

**Table 1:** Prominent Pin1 isomerization substrates and functional effects.

Substrate	Substrate Type	Pin1 Interaction	Proposed Pin1 Function
**Akt** **p70S6K**	PI3K pathway kinase	Stabilizes/activates	▪ Oncogenic dysregulation of downstream metabolic, proliferative, antiapoptotic pathway functions
**Cyclin D** **pRb**	G_1_/S activator G_1_/S inhibitor	Stabilizes/relocalizes Deactivates	▪ Increases checkpoint activation and cell cycle progression
**Pim1**	Oncogenic kinase	Destabilizes	▪ Blocks Pim1 antiapoptotic, cell cycle signaling, differentiation activity
**Raf1** **RSK2**	MAPK pathway kinases	+ Dephosphor/stabilizes + Phosphor/stabilizes	▪ Enhances AP-1 mediated transcription of Cyclin D
**SMAD**	Transactivator	Reduces protein levels	▪ Blocks TGF-β signaling
**Cdc25** **Incenp** **NIMA** **Survivin** **TopoIIα** **Wee1**	Mitotic regulators	Promotes dephosphor Unknown interaction Decreases activity Decreases protein levels Promotes phosphor Deactivates	▪ Regulates kinetics of mitosis progression and completion
**Centrosome**	Organelle	Enhances activity	▪ Promotes centrosome duplication prior to mitosis
**Histone H1**	Chromatin binding protein	+ Dephosphor/enhances binding	▪ Promotes chromatin binding, condensation, transcriptional repression
**Actin** **Tau**	Cytoskeletal proteins	Unknown interaction Promotes dephosphor	▪ Unknown function (actin); bound and incorporated into HIV-1 virions ▪ Limits abnormal microtubule/tangle formation, tauopathies (tau)
**KRMP1**	Kinesin-like motor	Unknown interaction	▪ Putative mitotic regulator and/or mitotic substrate transporter
**c-Myc**	TF	Enhances activity/destabilizes	▪ Promotes selective activation of cell proliferative/metabolic genes
**HDAC3**	Deacetylase	Destabilizes	▪ Promotes oncogenic transcriptional activation
**SMRT**	Transcriptional repressor	Destabilizes	▪ Blocks recruitment of HDACs to promoters, promotes transcription
**β-catenin**	TF	Stabilizes/activates	▪ Blocks repression, allowing Cyclin D upregulation
**Bcl2**	Antiapoptotic regulator	Destabilizes/deactivates	▪ Prevents inhibition of apoptosis
**c-Jun/c-Fos**	TFs	Stabilizes/activates	▪ Activates transcription through MAPK-AP-1 pathway
**p53**	DNA damage response TF	Stabilizes/activates	▪ Promotes apoptosis and cell cycle arrest
**p65 (NF-κB)**	TF	Relocalizes/stabilizes	▪ Prevents inhibition, activates angiogenic, antiapoptotic genes
**Notch1/NICD**	Growth factor receptor	Stimulates cleavage	▪ Promotes NICD release, downstream Notch signaling with RBP-Jk
**Hif-1** **BiP/Grp78**	Hypoxia regulator ER stress regulator	Upregulates expression	▪ Enhances HIF-1-mediated VEGF production, UPR activation
**APP**	Membrane protein	+ Dephosphor/destabilizes	▪ Prevents improper processing, accumulation of amyloid-β plaques
**ADAR2**	Adenosine deaminase	Stabilizes	▪ Promotes editing of GluR2 mRNA for calcium flux in neurons
**Nanog** **Oct4**	Self-renewal TFs	Stabilizes/enhances activity	▪ Represses differentiation of embryonic stem cells
**RNAP II CTD** **hSpt5**	Transcriptional regulators	Controls activity/relocalizes	▪ Regulates transcription termination, elongation, RNA processing, RNAP II storage
**TRF1**	Shelterin member	Destabilizes	▪ Prevents shortening of telomeres, telomere dysfunction
**APOBEC3G** **Capsid protein** **Integrase**	Cytidine deaminase HIV-1 virion protein HIV-1 enzyme	Inhibits activity Stabilizes Stabilizes	▪ Blocks HIV-1 restriction, promoting replication ▪ Promotes capsid uncoating ▪ Promotes HIV-1 incorporation into the host genome
**BALF5**	EBV polymerase catalytic subunit	Enhances activity	▪ Enhances viral replication
**Hbx**	HBV transactivator	Stabilizes	▪ Enhances HBV-induced hepatocarcinogenesis via signaling dysregulation
**IRF3**	IFN response regulator	Destabilizes homodimers	▪ Represses IFN innate antiviral response
**Tax**	HTLV-1 transactivator	Stabilizes/activates	▪ Enhances transcriptional activity and oncogenesis

**+ (De)phosphor **= promotes (de)phosphorylation. *Abbreviations and citations in text.*

### 3.3. Pin1 Has a Novel Role in KSHV Lytic Reactivation

Pin1 has been found to play a role in herpesviral pathogenesis as well. In HCMV infection, Pin1 is recruited to aid reorganize nuclear lamin A/C upon phosphorylation of the lamina by viral kinase pUL97 and cellular PKC [162]. Pin1 also associates with a number of proteins that could play roles in viral egress, including microtubule binding protein tau, actin filaments (which are known to be incorporated into virions during assembly) and, interestingly, kinesin-related protein KRMP1, a motor protein similar to kinesin or myosin that may play an important role, together with Pin1, in regulation of mitosis, potentially through the transport of Pin1 and other mitotic substrates [119,133,163,164]. And in a recent study by Narita *et al.*, Pin1 was found to bind to γ-herpesvirus EBV protein BALF5, the catalytic subunit of the viral DNA polymerase, and enhance EBV replication [165]. 

**Figure 4:**
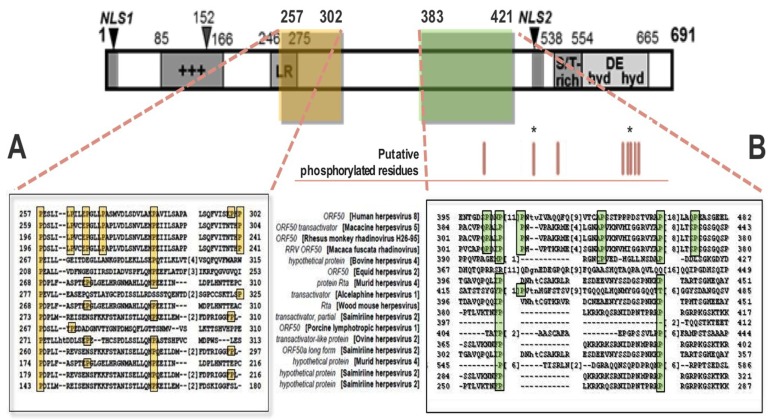
Rta protein is rich with conserved prolines. 17% of Rta’s conserved aa are prolines. At bottom are Rta primary sequence alignments of two proline-rich regions (**A**) and (**B**), denoted by yellow and green boxes, to the TAF50 *γ*-*herpesvirinae* superfamily. Numbers indicate aa position. +++ = positively-charged aa-rich, LR = leucine heptapeptide repeat domain, S/T = serine/threonine-rich, hyd DE hyd = hydrophobic/charged/hydrophobic aa-rich, NLS = nuclear localization signal sequence. Red lines mark putative phosphorylated residue sites; * = known phosphorylated residue. Figure modified from [166].

Recently, our lab investigated a putative interaction between KSHV Rta and PPIase Pin1 in the regulation of Rta-mediated lytic reactivation at multiple stages of the lytic cycle cascade. *In silico* alignment analysis of the transcription activation factor (TAF) 50 superfamily revealed that Rta homologs share a consensus of 134 amino acids (residues conserved between KSHV Rta and at least one additional member). Proline consists of 38 of these amino acids, accounting for 16.7% of all of Rta’s conserved residues (Figure 4) [40,166]. The high degree of conservation supported their putative functional significance. We reasoned that modification of prolines may regulate Rta’s efficiency in transactivating target genes, licensing viral DNA replication and interacting with protein partners—which could drive the latency-lytic cycle balance in favor of productive replication. 

In our report, we demonstrated an interaction between cellular isomerase Pin1 and KSHV lytic switch Rta. We showed that Pin1 is expressed and active in infected PEL cells after lytic cycle induction, and that Pin1 directly interacts with Rta *in vitro* and in infected cells, most likely at one of Rta’s putative conserved Pin1-recognition (pS/T-P) motifs [166]. Pin1 did not, however, interact with the essential Rta cofactor and Notch effector RBP-Jk by GST pulldown assay [166]. Pin1 enhanced Rta transactivation at two viral promoters in transient transfections [166]. Cotransfection of Rta with Pin1 appeared to result in enhanced redistribution of Rta from punctae to strong pan-nuclear expression in the majority of cells that coexpressed Pin1 (89%) [166]. This effect seemed to involve minute amounts of Pin1, as most coexpressed cells with Pin1 even modestly over background displayed Rta relocalization [166]. Overall Rta expression was also markedly stronger between punctae and pan-nuclear localization. In WT and Rta-inducible, virally-infected PEL, iSLK and Vero cells, we showed that Pin1 has a time-dependent effect on lytic reactivation, enhancing early-stage but inhibiting late-stage lytic cycle function [166]. Early-stage enhancement was shown via Rta-mediated DE transactivation and viral DNA-based experiments that overexpress or ablate Pin1. Late-stage inhibition by Pin1, meanwhile, was shown via reactivation experiments in WT and Rta-inducible PELs, in a Rta-inducible iSLK BAC16-based cell line system in which the viral allele of Rta is rendered defective by insertion of a stop codon (BAC16-RTAstop), and finally in a new, two-step KSHV reporter Vero-based cell line system developed by Gantt and colleagues that contain the secreted alkaline phosphatase (SeAP) gene under the control of an upstream tetracycline responsive element (TRE) promoter [167]. Together, those studies suggest that expression of late gene K8.1, and subsequent infectious virion release, are markedly inhibited [166,168].

Our published report showed that KSHV co-opts Pin1 function as a molecular timer, where by Pin1 enhances Rta expression, Rta transactivation activity at Rta-responsive promoters and Rta-mediated viral DNA replication, but inhibits late gene synthesis and virion production [166]. We propose that this dichotomy of Pin1 function can impart KSHV with a prosurvival, abortive lytic reactivation pathway, one which we hypothesize may regulate viral pathogenesis through the expression and activity of lytic cycle oncoproteins. To our knowledge, we mark the first discovery of an interaction between a DNA virus transcription factor and Pin1.

## 4. Significance of Convergence of Pin1 Function with Regulation of KSHV Lytic Reactivation

### 4.1. Ectopic Pin1 Is Sufficient to Induce Rta Expression: Putative Mechanisms

Early Pin1 activity may be important due to its sufficiency to induce Rta expression. Pin1 is known to affect protein expression in a number of ways. First, it could upregulate Rta transcription through well-described signaling pathways. Chief among them are c-Jun and c-Fos, which constitute the transcriptional regulator activating protein (AP)-1, as well as hypoxia inducible factor 1α (HIF-1α), an important regulator within the hypoxia response [89,92,169,170]. In PEL cells, HIF-1α is always active due to LANA inhibition of VHL [171]. HIF-1α can then activate Rta expression as well as Rta co-activators [69,89,90,172]. It is intriguing that Pin1 engages in a positive feedback mechanism with HIF-1α during the hypoxia response [146,147,173]. Additionally notable is that the ER stress response sensor BiP is upregulated by Pin1, an activity that is conserved with other PPIases in their role as protein folding regulators [174]. Meanwhile, AP-1 involvement in potential Pin1-dependent Rta transactivation has additional implications. Pin1 function upstream of Rta IE gene expression, and through the AP-1 pathway, is highly similar to TPA’s mechanism of lytic induction [81,134,151,152,175]. In Pin1 −/− MEFs, TPA induction of the AP-1 pathway was found to be much weaker than in Pin1 +/+ MEFs [175]. Second, Pin1 could affect transcriptional elongation and posttranscriptional splicing [115,128,137,176]. This could occur via Pin1's described interaction with RNA polymerase (RNAP) II [137,176,177]. Pin1 has been shown to play a role in control of carboxyl-terminal domain (CTD) phosphorylation [177]. The combination of phosphorylation and *cis*/*trans* isomerization designates the so-called “CTD code” that coordinates proteins involved in RNAP II-mediated events, including mRNA processing [128,177]. Future studies of Rta transcriptional and posttranscriptional control in the presence of Pin1, as well as Pin1 promoter binding and activity studies in infected PELs, will help confirm and define the above-suggested interplays.

### 4.2. Pin1 Directly Binds to Rta and Enhances Rta Transactivation

In our report, we found that Pin1 and Rta directly interact *in vitro* and in infected cell lysates. As with most of its protein substrates, Pin1 could enhance expression independently of its transcriptional effects through direct stabilization of Rta protein. Pin1 often stabilizes proteins that may otherwise be ubiquitylated and targeted for proteosomal degradation, such as Cyclin D [115,133,178]. The most applicable examples of this are the viral Tax and HBx oncoproteins and transcription factors of HTLV-1 and hepatitis B virus (HBV), respectively, as previously discussed [159,161]. These factors are prevented from degradation by conformational changes induced due to prolyl isomerization, allowing their protein levels to accumulate for efficient downstream activities, including transactivation and productive viral replication. It is possible that Pin1 could affect Rta stability by either inhibiting Rta auto-ubiquitylation via Rta’s E3 ubiquitin ligase domain, or by enhancing Rta-induced degradation, also through its E3 ligase activity, of Rta repressors such as K-RBP and IRF7 [63,64,68]. Indeed, these repressors bind to Rta within proline-rich regions containing putative Pin1 isomerization motifs; Pin1 activity could prevent their association.

As we observed that the Pin1 interaction appeared to be stronger with full-length WT Rta, we cannot rule out that Pin1 has multiple binding motifs within Rta. First, Pin1 is known to bind to a number of its targets at more than one motif; this includes p53, c-Jun, Nanog and Akt [126,133,134,178,179,180]. Second, Pin1 binding and isomerization activities are separable and complex. Binding motifs can be recognized by each domain and acted upon with different specificities [111,115,116,123,130,181]. In other words, Pin1 can bind one motif, but very well isomerize another.

**Figure 5:**
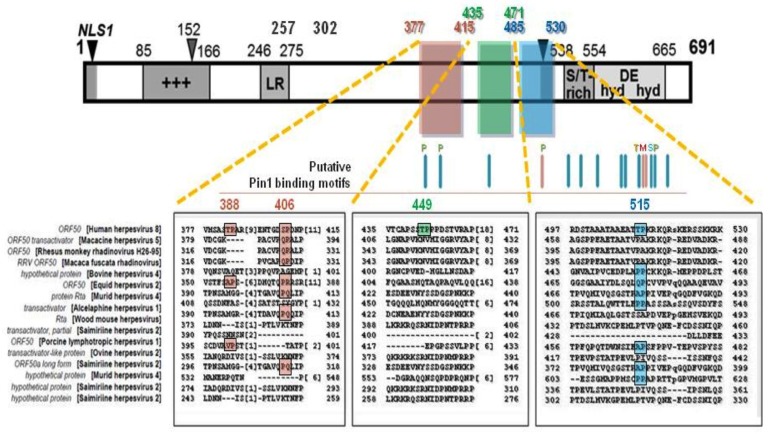
Rta protein has putative, conserved Pin1 motifs. Blue bars indicate 15 putative S/T-P motifs, sites for Pin1 binding and isomerization, which are characterized by a phosphoserine or phosphothreonine immediately preceding a proline. Letters indicate aa that are parts of possible Pin1 motifs that are phylogenetically conserved between KSHV Rta and at least one additional *γ-herpesvirinae* family member ORF50 homolog (part of the TAF50 protein superfamily). Motif bars colored in red indicate those that are known to be phosphorylated (T515, S634 and S636; see Figure 4). Putative Pin1 motifs T449, T540, T628, S644 and S650 are also putative phosphorylated residues. Boxes at bottom show alignments of proline-rich and RBP-JK binding regions of Rta. Numbers indicate aa position. +++ = positively-charged aa-rich, LR = leucine heptapeptide repeat domain, S/T = serine/threonine-rich, hyd DE hyd = hydrophobic/charged/hydrophobic aa-rich, NLS = nuclear localization signal sequence, P = conserved proline, S = conserved serine, T = conserved threonine, M = fully conserved S/T-P motif. Figure modified from [166].

Other possible motifs could also have binding and/or isomerization specificity, including Thr449, Thr515, Ser634 (which is a fully conserved Pin1 motif) and Ser636 (Figure 5) [166]. The latter three, in this case, are interesting putative motifs, as they are the only known potential Pin1 sites to date that has been previously shown to be phosphorylated in *in vitro* binding assays or in infected cell-based Western blots, by Rta transactivation inhibitor hKFC (for Thr515) and CDK9 (Ser634/636), respectively (and it bears mentioning that the other, putative CDK binding sites, at Thr449, Thr540, Thr628, Ser644 and Ser650, are also all putative Pin1 sites) [42,44,45]. CDK9 kinase activity on Rta is notable because CDK9 is a catalytic subunit of positive transcription elongation factor b (P-TEFb), which associates with the promoter-paused RNAP II’s CTD and activates transcriptional elongation [44]. Rta likely recruits CDK9 to viral promoters, where the kinase licenses transcription as well as positively regulates Rta activity. Pin1, which as previously discussed, binds to the CTD and regulates transcriptional elongation, is known to bind to CDK9-phosphorylated substrate hSpt5, an elongation inhibitor [137,182]. Thus, Pin1 interactions with both CDK9 targets, hSpt5 and Rta, could enhance RNAP II transcriptional elongation at KSHV gene promoters. While it is tempting to suggest that these could represent *bona fide* pS/T-P motifs, the cytosolic localization of hKFC makes an Rta‑regulating Pin1 modification through hKFC in infected cells suspect, while for CDK9, in light of the lack of Pin1 binding to the Rta aa 525–691 truncation mutant, a true Pin1 motif beyond aa 525 appears less likely [45,166]. Nevertheless, Pin1 binding at Ser634/636 in infected cells, alone or combined, could still be possible, and future functional binding analysis with Pin1 motif mutants of Rta will be required to clarify this interaction. Taking into account the intricate regulation by Pin1 reported throughout this review, multiple binding sites could provide Pin1 with a combinatorial influence on Rta function, much as Pin1 coordinates the CTD code of RNAP II, based directly on the phosphorylation status of certain motifs. This control could be one explanation for the divergent effects of Pin1 during the lytic cycle, which will be further addressed below.

Pin1 binding to Rta could mediate a variety of different effects on Rta beyond stability, including tetramerization. Bearing in mind the importance of conserved prolines on Rta higher-order structure, that Pin1 binding to the Rta aa 170–400 region overlaps with the tetramerization and proline-rich domains (Figure 1), the putative Pin1 motif at Thr388 (Figure 5) and that previous data showed that RBP-Jk also binds to Rta aa 170–400 in solution [47,48], it is reasonable to suggest that Pin1 binding to Rta could regulate Rta’s tetramer formation, allowing it to interact with RBP-Jk and transactivate downstream viral and cellular genes [166].

In the literature, Pin1 has been previously reported to affect protein multimerization, in that case destabilizing IRF3 homodimer formation [157]. Pin1 also has a known role in upregulating Notch1 activity through enhanced cleavage of NICD by γ-secretase, which it could perhaps do in KSHV-infected cells in concert with the reported stabilization of NICD by LANA or activation of Notch4 by vGPCR [101,147,183]. NICD can weakly bind to RBP-Jk alone for modest transactivation at viral promoters, but can act synergistically with RtaΔSTAD due to the effect of NICD’s transactivation domain [47].

It is important to point out that, although we tested and confirmed that Pin1 greatly enhanced Rta-mediated transactivation and DE gene expression using well-studied promoter markers nut-1 and Mta, we did not directly observe Pin1’s effect on KSHV’s lytic cycle oncoproteins, such as vGPCR or vIL-6, *etc.* Therefore, while our data suggest that Pin1 affects DE gene expression nonspecifically, we cannot make concrete conclusions. The same is true in regards to Pin1’s effect on KSHV oncogenic properties, including VEGF production and angiogenesis, cell cycle disruption and apoptotic subversion. Could Pin1 expression and/or activity distinguish Rta-initiated reactivation cascades that differ in expression of replication protein from oncoproteins? If so, could Pin1 activity determine the oncogenicity of KSHV infection? As the purpose of our investigation was to identify and characterize the qualitative role of Pin1 on the Rta lytic switch and on lytic reactivation, further studies addressing such questions will develop Pin1’s lytic cycle-based molecular mechanisms as well as the broader phenotypic and tumorigenic ramifications of these, and other, activities at multiple stages of the KSHV life cycle, including in *de novo* infection and egress.

The mechanism of how, exactly, Pin1 strengthens Rta-mediated transactivation and DE gene expression is unclear and the subject of ongoing examination. Additional, indirect enhancement of Rta transactivation efficiency could be aided by Pin1’s function with RNAP II in transcriptional elongation or termination, or in posttranscriptional processing and splicing [128,137]. These functions, especially the latter, could cooperate with Mta. Like Pin1, Mta is enriched at nuclear speckles and is involved in posttranscriptional elongation and cellular factor-dependent viral pre-mRNA splicing [95,96,184]. Mta also stabilizes nascent viral transcripts and facilitates export of intronless viral transcripts, which account for ~70% of all KSHV mRNAs [95,96,184]. Pin1 and Mta could together coordinate RNAP II transcriptional progression in conjunction with processing factors stored in nuclear speckles, with Mta and Pin1 first directly enhancing Rta function at promoters, followed by Mta stabilizing elongating transcripts, Pin1 enabling proper termination via CTD code modification and then Mta shuttling intronless mRNAs out of the nucleus for efficient translation. Taken together, along with the context-dependent Pin1 motifs scenario, Pin1 could be part of a multi-tiered regulatory loop consisting of different functional consequences for Rta expression and for Rta transactivation. 

### 4.3. Pin1 Enhances KSHV Lytic DNA Replication

Results from our report also suggested that inhibition of Pin1 drastically reduces the rate of replication as it proceeds through the lytic cycle. This could simply be due to functional carryover from reduced DE gene synthesis. However, the strong impact of juglone on replication suggests that loss of Pin1 may have a broader effect than transactivation alone. We can further suggest, then, that Pin1’s transactivation enhancement of Rta could extend to Rta’s association at oriLyts and its role in lytic replication in concert with K-bZIP [7,37,61]. Pin1 binding to Rta could enhance Rta’s ability to recognize its elements at oriLyts, to recruit basal DNA replication factors in a manner analogous to Rta’s recruitment of transcription factors at viral promoters, or to interact with K-bZIP directly. Pin1’s effects on Rta-mediated transactivation and replication are probably based on a single modification that simultaneously enhances both processes, although additional work will need to be done to rule out a more complex regulatory mechanism. 

### 4.4. Pin1 Represses KSHV Late Gene Expression and Virion Production

We expected Pin1 to continue to act as an Rta enhancer and upregulate productive lytic reactivation as assessed by release of infectious virus, which often positively correlates to viral replication. Instead, data from our report strongly suggested that Pin1 inhibits virion production [166]. Rather than acting as a positive cofactor that enhances Rta-mediated transactivation and replication, Pin1 may actually be a complex, bimodal regulator of lytic reactivation, as it later acts as a negative cofactor that represses virion production and release.

A number of potential hypotheses could be proposed that address the manner in which Pin1 inhibits late gene synthesis following Pin1’s enhancement of viral DNA replication. Pin1, despite its oncogenic functions, also has interactions with a variety of cellular or viral regulators that could negatively impact lytic cycle progression. One intriguing explanation for Pin1’s repression of late gene synthesis comes from its potential cooperation, as discussed above, with Mta during posttranscriptional and splicing regulation. Mta binds to intronless viral mRNA for efficient export into the cytosol for translation [95,96]. The cellular polyadenylation-binding protein C1 (PABPC1), with a natural localization in the cytosol, protects polyA transcripts from cytidine deadenylases with nanomolar affinity and enhances both mRNA nuclear export and translational initiation [60,96]. Upon expression of the lytic cycle, viral shutoff exonuclease (vSOX) relocalizes PABPC1 to the nucleus, where it binds and stabilizes cellular and viral polyA mRNA as before, but sequesters these transcripts in the nucleus [60,96]. As Mta binding to intronless viral mRNA cannot overcome PABPC1 sequestration, this would, in effect, shut down all polyA transcript export and translation (which, recall, accounts for 70% of all viral mRNA) [60,96]. However, nut-1/PAN, the DE noncoding transcript (at 500,000 copies per virally-reactivated PEL cell, by far the most abundant, at upward of 80% of all cellular polyA mRNA), has been found to bind to PABPC1 and titrate the protein away from polyA mRNA; since Mta binds only intronless viral mRNAs, it was hypothesized that the cooperation of both nut-1 and Mta allows for preferential nuclear export of viral transcripts, while PABPC1 still drastically slows the export of cellular transcripts [59,60,96]. Thus, the vast majority of protein produced during lytic reactivation is viral. Notably, however, the loss of nut-1 accumulation has been observed to cause deficient late gene synthesis, with concomitant loss of virion production, even despite normal Rta expression, transactivation and viral DNA replication [60]. 

As Pin1 is involved in many of the same processes as Mta, one could postulate that Pin1 may interfere in some way with PABPC1 protein interaction with nut-1 in the nucleus following vSOX activation; this would achieve the same functional result as nut-1 deficiency, since inhibition of nut-1 expression does not appear to occur based on our transactivation and DE gene expression-based data. This modulation of nut-1 would prevent late gene synthesis without impacting any previous lytic cycle stage nor the suppression of cellular gene translation. The scenario also allows for a much simpler mechanism of bimodal Pin1 function without the requirement for any direct, mid-lytic cycle regulatory alteration of Pin1 activity, although combinatorial Pin1 binding to Rta could very well still occur. Nevertheless, Pin1’s dynamic regulation could be “built in” to the lytic cycle program, one in which enactment of a single modification for each of a limited number of viral factors, particularly Rta, could achieve a complex, tightly-coordinated progression of events. Accounting for such stage-specificity, Pin1 blockade of late gene synthesis following Rta induction could be due to potential time-dependent cofactors, such as particular DE protein interaction with Pin1; unknown differences at late gene promoters that prohibit Rta transactivation in concert with Pin1; upregulation of cellular or viral factors following the onset or completion of viral DNA replication; or aforementioned combinatorial Pin1 activity that directly affects Rta function. 

### 4.5. Molecular Timing Model for Pin1’s Effects on KSHV Replication and Pathogenesis

Taking the culmination of results from our report together, each of the above possibilities could allow for Pin1 to function in a divergent manner as a postreplication lytic cycle inhibitor capable of reducing the efficiency of, or altogether counteracting, prolonged virion release and cell lysis. Put another way, Pin1 is co-opted by KSHV to regulate the timeframe of reactivation and the balance between abortive and productive lytic reactivation. Pin1 overexpression may shift this balancing act in favor of repression, while too weak Pin1 signaling might not activate Rta expression to begin with. Thus, a “Goldilocks” level of Pin1 regulatory activity during the lytic cycle may be required for the cycle’s initiation, progression and completion, a role that possibly evolved as a prosurvival, immunoevasive measure that emphasizes DE gene expression and clandestine viral replication within an infected cell population. 

This was in keeping with a dosage-dependent timing mechanism that may allow for only a subset of cells induced for Rta expression to advance through the full lytic cycle. Indeed, reactivation occurs in a small subpopulation of KSHV-infected tumor cells, produces oncogenic DE gene products that are believed to be essential for tumor growth and is governed by inefficient Rta activity as the vast majority of Rta-expressing cells do not coexpress late gene markers, such as K8.1 [4,7,50]. Molecular hijacking of a conserved cellular timer that has subtle, but powerful effects on a variety of oncogenic and pro-viral processes could have evolved as a protective rheostat that minimizes noise for lytic switch induction. Fine-tuning the ratios between latency, and abortive and productive replication, could mitigate spurious and potentially self-limiting “runaway” virion production, host cell lysis and immune system activation that disrupt the local infected cell microenvironment, prevent longterm cellular stability of viral episomes and risk overall virus survival in the host. 

**Figure 6:**
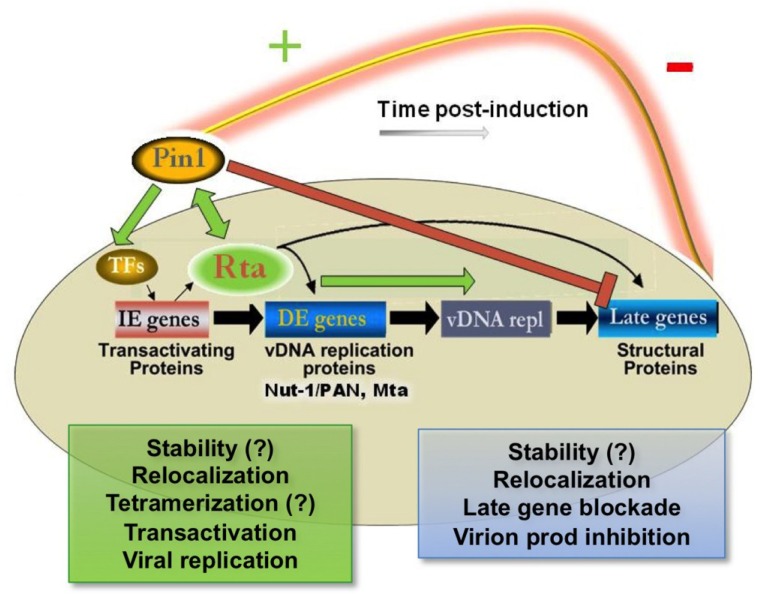
Pin1 acts a novel KSHV lytic cycle timer through regulation of Rta expression and downstream activity. Disease models of cells infected with latent KSHV show that a small subpopulation undergo reactivation, which is thought to promote tumor growth. The lytic cycle cascade begins with Rta and other immediate-early (IE) protein expression, followed by Rta-mediated transactivation of delayed-early (DE) genes, such as Mta, and which include DE oncoproteins and viral DNA replication factors. Upon completion of Rta-dependent viral replication, late gene synthesis proceeds with structural and glycoprotein expression, such as K8.1. Finally, assembly and egress of infectious virions allows for dissemination within the host and to other individuals. It is likely that Pin1 isomerase modulates Rta activity during reactivation. During early reactivation events (Rta DE transactivation, viral replication), Pin1 strongly enhances Rta function. However, by an unknown mechanism within the lytic cascade, Pin1 transitions into an inhibitor of late events (late gene synthesis, infectious virus release), halting productive reactivation. Thus, Pin1 functions as a molecular timer. Pin1 is known to control strength and duration of an array of normal and pathological cellular signals, and we believe Pin1's timing activity is co-opted by KSHV to allow for an evolutionarily-advantageous, nonproductive window allowing for DE gene expression while protecting against cell lysis and immune response activation. Figure modified from [166].

We proposed a dynamic molecular timing model in which Pin1 upregulates Rta expression, transactivation and viral replication *ab initio*, but then later suppresses optimal, productive lytic replication (Figure 6). If proven correct, by opening up a threshold- or kinetics-dependent “window” that licenses the initiation and progression of reactivation, KSHV has evolved an exquisitely balanced, prosurvival lytic program: co-option of a multifunctional cellular timer, Pin1 isomerase, maximizes Rta-mediated transactivation of viral lytic cycle genes, and then conversely protects against unchecked infectious virion production that would otherwise compromise host cell integrity and viral immune evasion for the vast majority of KSHV-infected cells that may reside within tumors. Our findings therefore point to Pin1 as an attractive antiherpesviral drug candidate that could be of potentially efficacious use in the treatment of HIV-1-positive and immunocompromised patients at risk for, or afflicted with, KSHV-derived malignancies. 

## References

[1] Neipel F., Albrecht J.C., Fleckenstein B. (1997). Cell-homologous genes in the Kaposi’s sarcoma-associated rhadinovirus human herpesvirus 8: Determinants of its pathogenicity?. J. Virol..

[2] Dourmishev L.A., Dourmishev A.L., Palmeri D., Schwartz R.A., Lukac D.M. (2003). Molecular genetics of Kaposi’s sarcoma-associated herpesvirus (human herpesvirus-8) epidemiology and pathogenesis. Microbiol. Mol. Biol. Rev..

[3] Edelman D.C. (2005). Human herpesvirus 8—A novel human pathogen. Virol. J..

[4] Ganem D., Knipe D.M., Howley P.M. (2007). Kaposi’s sarcoma-associated herpesvirus. Fields Virology.

[5] Wen K.W., Damania B. (2010). Kaposi sarcoma-associated herpesvirus (KSHV): molecular biology and oncogenesis. Cancer Lett..

[6] Hengge U.R., Ruzicka T., Tyring S.K., Stuschke M., Roggendorf M., Schwartz R.A., Seeber S. (2002). Update on Kaposi’s sarcoma and other HHV8 associated diseases. Part 1: Epidemiology, environmental predispositions, clinical manifestations, and therapy. Lancet Infect. Dis..

[7] Lukac D.M., Yuan Y., Arvin A., Campadelli-Fiume G., Mocarski E., Moore P.S., Roizman B., Whitley R., Yamanishi K. (2007). Reactivation and lytic replication of KSHV. Human Herpesviruses: Biology, Therapy, and Immunoprophylaxis.

[8] Ruocco E., Ruocco V., Tornesello M.L., Gambardella A., Wolf R., Buonaguro F.M. (2013). Kaposi's sarcoma: Etiology and pathogenesis, inducing factors, causal associations, and treatments: facts and controversies. Clin. Dermatol..

[9] Sathish N., Wang X., Yuan Y. (2012). Tegument Proteins of Kaposi’s Sarcoma-Associated Herpesvirus and Related Gamma-Herpesviruses. Front. Microbiol..

[10] Greene W., Kuhne K., Ye F., Chen J., Zhou F., Lei X., Gao S.J. (2007). Molecular biology of KSHV in relation to AIDS-associated oncogenesis. Cancer Treat. Res..

[11] Hengge U.R., Ruzicka T., Tyring S.K., Stuschke M., Roggendorf M., Schwartz R.A., Seeber S. (2002). Update on Kaposi’s sarcoma and other HHV8 associated diseases. Part 2: Pathogenesis, Castleman’s disease, and pleural effusion lymphoma. Lancet Infect. Dis..

[12] Chen Y.B., Rahemtullah A., Hochberg E. (2007). Primary effusion lymphoma. Oncologist.

[13] Noguchi K., Fukazawa H., Murakami Y., Takahashi N., Yamagoe S., Uehara Y. (2007). Gamma-herpesviruses and cellular signaling in AIDS-associated malignancies. Cancer Sci..

[14] Ye F., Lei X., Gao S.J. (2011). Mechanisms of Kaposi’s Sarcoma-Associated Herpesvirus Latency and Reactivation. Adv. Virol..

[15] Dittmer D.P., Damania B. (2013). Kaposi sarcoma associated herpesvirus pathogenesis (KSHV)—an update. Curr. Opin. Virol..

[16] Scholz B.A., Harth-Hertle M.L., Malterer G., Haas J., Ellwart J., Schulz T.F., Kempkes B. (2013). Abortive lytic reactivation of KSHV in CBF1/CSL deficient human B cell lines. PLoS Pathog..

[17] Sun R., Lin S.-F., Staskus K., Gradoville L., Grogan E., Haase A., Miller G. (1999). Kinetics of Kaposi's sarcoma-associated herpesvirus gene expression. J. Virol..

[18] Sternbach G., Varon J. (1995). Moritz Kaposi: Idiopathic pigmented sarcoma of the skin. J. Emerg. Med..

[19] Rezza G., Lennette E.T., Giuliani M., Pezzotti P., Caprilli F., Monini P., Butto S., Lodi G., Di Carlo A., Levy J.A., Ensoli B. (1998). Prevalence and determinants of anti-lytic and anti-latent antibodies to human herpesvirus-8 among Italian individuals at risk of sexually and parenterally transmitted infections. Int. J. Cancer.

[20] Lu F., Zhou J., Wiedmer A., Madden K., Yuan Y., Lieberman P.M. (2003). Chromatin remodeling of the Kaposi’s sarcoma-associated herpesvirus ORF50 promoter correlates with reactivation from latency. J. Virol..

[21] Pantry S.N., Medveczky P.G. (2009). Epigenetic regulation of Kaposi’s sarcoma-associated herpesvirus replication. Semin. Cancer Biol..

[22] Sathish N., Zhu F.X., Yuan Y. (2009). Kaposi’s sarcoma-associated herpesvirus ORF45 interacts with kinesin-2 transporting viral capsid-tegument complexes along microtubules. PLoS Pathog..

[23] Subramanian R., Sehgal I., D'Auvergne O., Kousoulas K.G. (2010). Kaposi’s sarcoma-associated herpesvirus glycoproteins B and K8.1 regulate virion egress and synthesis of vascular endothelial growth factor and viral interleukin-6 in BCBL-1 cells. J. Virol..

[24] Montaner S., Sodhi A., Molinolo A., Bugge T.H., Sawai E.T., He Y., Li Y., Ray P.E., Gutkind J.S. (2003). Endothelial infection with KSHV genes *in vivo* reveals that vGPCR initiates Kaposi’s sarcomagenesis and can promote the tumorigenic potential of viral latent genes. Cancer Cell.

[25] Liang C., Lee J.S., Jung J.U. (2008). Immune evasion in Kaposi’s sarcoma-associated herpes virus associated oncogenesis. Semin. Cancer Biol..

[26] Areste C., Blackbourn D.J. (2009). Modulation of the immune system by Kaposi’s sarcoma-associated herpesvirus. Trends Microbiol..

[27] Damania B. (2004). Oncogenic gamma-herpesviruses: Comparison of viral proteins involved in tumorigenesis. Nat. Rev. Microbiol..

[28] Mutlu A.D., Cavallin L.E., Vincent L., Chiozzini C., Eroles P., Duran E.M., Asgari Z., Hooper A.T., La Perle K.M., Hilsher C., Gao S.J., Dittmer D.P., Rafii S., Mesri E.A. (2007). *In vivo*-restricted and reversible malignancy induced by human herpesvirus-8 KSHV: A cell and animal model of virally induced Kaposi's sarcoma. Cancer Cell.

[29] Montaner S., Sodhi A., Ramsdell A.K., Martin D., Hu J., Sawai E.T., Gutkind J.S. (2006). The Kaposi’s sarcoma-associated herpesvirus G protein-coupled receptor as a therapeutic target for the treatment of Kaposi’s sarcoma. Cancer Res..

[30] Zong J.C., Ciufo D.M., Alcendor D.J., Wan X., Nicholas J., Browning P.J., Rady P.L., Tyring S.K., Orenstein J.M., Rabkin C.S. (1999). High-level variability in the ORF-K1 membrane protein gene at the left end of the Kaposi’s sarcoma-associated herpesvirus genome defines four major virus subtypes and multiple variants or clades in different human populations. J. Virol..

[31] Polson A.G., Wang D., DeRisi J., Ganem D. (2002). Modulation of host gene expression by the constitutively active G protein-coupled receptor of Kaposi’s sarcoma-associated herpesvirus. Cancer Res..

[32] Lagunoff M., Lukac D.M., Ganem D. (2001). Immunoreceptor tyrosine-based activation motif-dependent signaling by Kaposi’s sarcoma-associated herpesvirus K1 protein: Effects on lytic viral replication. J. Virol..

[33] Moore P.S., Boshoff C., Weiss R.A., Chang Y. (1996). Molecular mimicry of human cytokine and cytokine response pathway genes by KSHV. Science.

[34] Lukac D.M., Renne R., Kirshner J.R., Ganem D. (1998). Reactivation of Kaposi’s sarcoma-associated herpesvirus infection from latency by expression of the ORF 50 transactivator, a homolog of the EBV R protein. Virology.

[35] Lukac D.M., Kirshner J.R., Ganem D. (1999). Transcriptional activation by the product of open reading frame 50 of Kaposi's sarcoma-associated herpesvirus is required for lytic viral reactivation in B cells. J. Virol..

[36] Xu Y., AuCoin D.P., Huete A.R., Cei S.A., Hanson L.J., Pari G.S. (2005). A Kaposi’s sarcoma-associated herpesvirus/human herpesvirus 8 ORF50 deletion mutant is defective for reactivation of latent virus and DNA replication. J. Virol..

[37] Guito J., Lukac D.M. (2012). KSHV Rta Promoter Specification and Viral Reactivation. Front. Microbiol..

[38] Chen J., Ye F., Xie J., Kuhne K., Gao S.J. (2009). Genome-wide identification of binding sites for Kaposi’s sarcoma-associated herpesvirus lytic switch protein, RTA. Virology.

[39] Carroll K.D., Khadim F., Spadavecchia S., Palmeri D., Lukac D.M. (2007). Direct interactions of Kaposi's sarcoma-associated herpesvirus/human herpesvirus 8 ORF50/Rta protein with the cellular protein octamer-1 and DNA are critical for specifying transactivation of a delayed-early promoter and stimulating viral reactivation. J. Virol..

[40] Damania B., Jeong J.H., Bowser B.S., DeWire S.M., Staudt M.R., Dittmer D.P. (2004). Comparison of the Rta/Orf50 transactivator proteins of gamma-2-herpesviruses. J. Virol..

[41] Kieff E., Rickinson A.B., David K.M. (2007). Epstein-Barr virus and its replication. Fields Virology.

[42] Campbell M., Izumiya Y. (2012). Post-Translational Modifications of Kaposi’s Sarcoma-Associated Herpesvirus Regulatory Proteins—SUMO and KSHV. Front. Microbiol..

[43] Ko Y.C., Tsai W.H., Wang P.W., Wu I.L., Lin S.Y., Chen Y.L., Chen J.Y., Lin S.F. (2012). Suppressive regulation of KSHV RTA with O-GlcNAcylation. J. Biomed. Sci..

[44] Tsai W.H., Wang P.W., Lin S.Y., Wu I.L., Ko Y.C., Chen Y.L., Li M., Lin S.F. (2012). Ser-634 and Ser-636 of Kaposi’s Sarcoma-Associated Herpesvirus RTA are Involved in Transactivation and are Potential Cdk9 Phosphorylation Sites. Front. Microbiol..

[45] Gwack Y., Nakamura H., Lee S.H., Souvlis J., Yustein J.T., Gygi S., Kung H.J., Jung J.U. (2003). Poly(ADP-ribose) polymerase 1 and Ste20-like kinase hKFC act as transcriptional repressors for gamma-2 herpesvirus lytic replication. Mol. Cell Biol..

[46] West J.T., Wood C. (2003). The role of Kaposi’s sarcoma-associated herpesvirus/human herpesvirus-8 regulator of transcription activation (RTA) in control of gene expression. Oncogene.

[47] Carroll K.D., Bu W., Palmeri D., Spadavecchia S., Lynch S.J., Marras S.A., Tyagi S., Lukac D.M. (2006). Kaposi's Sarcoma-associated herpesvirus lytic switch protein stimulates DNA binding of RBP-Jk/CSL to activate the Notch pathway. J. Virol..

[48] Liang Y., Chang J., Lynch S., Lukac D.M., Ganem D. (2002). The lytic switch protein of KSHV activates gene expression via functional interaction with RBP-Jk, the target of the Notch signaling pathway. Genes Dev..

[49] Liang Y., Ganem D. (2003). Lytic but not latent infection by Kaposi’s sarcoma-associated herpesvirus requires host CSL protein, the mediator of Notch signaling. Proc. Natl. Acad. Sci. USA.

[50] Palmeri D., Spadavecchia S., Carroll K.D., Lukac D.M. (2007). Promoter- and cell-specific transcriptional transactivation by the Kaposi’s sarcoma-associated herpesvirus ORF57/Mta protein. J. Virol..

[51] Papugani A., Coleman T., Jones C., Zhang L. (2008). The interaction between KSHV RTA and cellular RBP-Jkappa and their subsequent DNA binding are not sufficient for activation of RBP-Jkappa. Virus Res..

[52] Gwack Y., Byun H., Hwang S., Lim C., Choe J. (2001). CREB-binding protein and histone deacetylase regulate the transcriptional activity of Kaposi’s sarcoma-associated herpesvirus open reading frame 50. J. Virol..

[53] Lukac D.M., Garibyan L., Kirshner J.R., Palmeri D., Ganem D. (2001). DNA binding by Kaposi’s sarcoma-associated herpesvirus lytic switch protein is necessary for transcriptional activation of two viral delayed early promoters. J. Virol..

[54] Bu W., Carroll K.D., Palmeri D., Lukac D.M. (2007). Kaposi’s sarcoma-associated herpesvirus/human herpesvirus 8 ORF50/Rta lytic switch protein functions as a tetramer. J. Virol..

[55] Palmeri D., Carroll K.D., Gonzalez-Lopez O., Lukac D.M. (2011). Kaposi’s sarcoma-associated herpesvirus Rta tetramers make high-affinity interactions with repetitive DNA elements in the Mta promoter to stimulate DNA binding of RBP-Jk/CSL. J. Virol..

[56] Ziegelbauer J., Grundhoff A., Ganem D. (2006). Exploring the DNA binding interactions of the Kaposi’s sarcoma-associated herpesvirus lytic switch protein by selective amplification of bound sequences *in vitro*. J. Virol..

[57] Bu W., Palmeri D., Krishnan R., Marin R., Aris V.M., Soteropoulos P., Lukac D.M. (2008). Identification of direct transcriptional targets of the Kaposi’s sarcoma-associated herpesvirus Rta lytic switch protein by conditional nuclear localization. J. Virol..

[58] Song M.J., Li X., Brown H.J., Sun R. (2002). Characterization of interactions between RTA and the promoter of polyadenylated nuclear RNA in Kaposi’s sarcoma-associated herpesvirus/human herpesvirus 8. J. Virol..

[59] Sun R., Lin S., Gradoville L., Miller G. (1996). Polyadenylated nuclear RNA encoded by Kaposi’s sarcoma-associated herpesvirus. Proc. Natl. Acad. Sci. USA.

[60] Borah S., Darricarrere N., Darnell A., Myoung J., Steitz J.A. (2011). A viral nuclear noncoding RNA binds re-localized poly(A) binding protein and is required for late KSHV gene expression. PloS Pathog..

[61] Wang Y., Li H., Chan M.Y., Zhu F.X., Lukac D.M., Yuan Y. (2004). Kaposi’s sarcoma-associated herpesvirus ori-Lyt-dependent DNA replication: *Cis*-acting requirements for replication and ori-Lyt-associated RNA transcription. J. Virol..

[62] Gwack Y., Hwang S., Byun H., Lim C., Kim J.W., Choi E.J., Choe J. (2001). Kaposi’s sarcoma-associated herpesvirus open reading frame 50 represses p53-induced transcriptional activity and apoptosis. J. Virol..

[63] Yang Z., Wood C. (2007). The transcriptional repressor K-RBP modulates RTA-mediated transactivation and lytic replication of Kaposi’s sarcoma-associated herpesvirus. J. Virol..

[64] Yang Z., Yan Z., Wood C. (2008). Kaposi’s sarcoma-associated herpesvirus transactivator RTA promotes degradation of the repressors to regulate viral lytic replication. J. Virol..

[65] Yang Z., Wen H.J., Minhas V., Wood C. (2009). The zinc finger DNA-binding domain of K-RBP plays an important role in regulating Kaposi’s sarcoma-associated herpesvirus RTA-mediated gene expression. Virology.

[66] Yu Y., Wang S.E., Hayward G.S. (2005). The KSHV immediate-early transcription factor RTA encodes ubiquitin E3 ligase activity that targets IRF7 for proteosome-mediated degradation. Immunity.

[67] Izumiya Y., Lin S.F., Ellison T., Chen L.Y., Izumiya C., Luciw P., Kung H.J. (2003). Kaposi's sarcoma-associated herpesvirus K-bZIP is a coregulator of K-Rta: physical association and promoter-dependent transcriptional repression. J. Virol..

[68] Gould F., Harrison S.M., Hewitt E.W., Whitehouse A. (2009). Kaposi’s sarcoma-associated herpesvirus RTA promotes degradation of the Hey1 repressor protein through the ubiquitin proteasome pathway. J. Virol..

[69] Cai Q., Lan K., Verma S.C., Si H., Lin D., Robertson E.S. (2006). Kaposi’s sarcoma-associated herpesvirus latent protein LANA interacts with HIF-1 alpha to upregulate RTA expression during hypoxia: Latency control under low oxygen conditions. J. Virol..

[70] Verma S.C., Lan K., Robertson E. (2007). Structure and function of latency-associated nuclear antigen. Curr. Top. Microbiol. Immunol..

[71] Ballestas M.E., Kaye K.M. (2011). The latency-associated nuclear antigen, a multifunctional protein central to Kaposi’s sarcoma-associated herpesvirus latency. Future Microbiol..

[72] Lan K., Kuppers D.A., Robertson E.S. (2005). Kaposi’s sarcoma-associated herpesvirus reactivation is regulated by interaction of latency-associated nuclear antigen with recombination signal sequence-binding protein Jkappa, the major downstream effector of the Notch signaling pathway. J. Virol..

[73] Li Q., Zhou F., Ye F., Gao S.J. (2008). Genetic disruption of KSHV major latent nuclear antigen LANA enhances viral lytic transcriptional program. Virology.

[74] Shin H.J., DeCotiis J., Giron M., Palmeri D., Lukac D.M. (2014). Histone deacetylase classes I and II regulate Kaposi’s sarcoma-associated herpesvirus reactivation. J. Virol..

[75] Qin Z., Jakymiw A., Findlay V., Parsons C. (2012). KSHV-Encoded MicroRNAs: Lessons for Viral Cancer Pathogenesis and Emerging Concepts. Int. J. Cell Biol..

[76] Samols M.A., Skalsky R.L., Maldonado A.M., Riva A., Lopez M.C., Baker H.V., Renne R. (2007). Identification of cellular genes targeted by KSHV-encoded microRNAs. PLoS Pathog..

[77] Ganem D., Ziegelbauer J. (2008). MicroRNAs of Kaposi’s sarcoma-associated herpes virus. Semin. Cancer Biol..

[78] Bellare P., Ganem D. (2009). Regulation of KSHV lytic switch protein expression by a virus-encoded microRNA: An evolutionary adaptation that fine-tunes lytic reactivation. Cell Host Microbe.

[79] Ye F.C., Zhou F.C., Xie J.P., Kang T., Greene W., Kuhne K., Lei X.F., Li Q.H., Gao S.J. (2008). Kaposi’s sarcoma-associated herpesvirus latent gene vFLIP inhibits viral lytic replication through NF-kappaB-mediated suppression of the AP-1 pathway: A novel mechanism of virus control of latency. J. Virol..

[80] Izumiya Y., Izumiya C., Hsia D., Ellison T.J., Luciw P.A., Kung H.J. (2009). NF-kappaB serves as a cellular sensor of Kaposi’s sarcoma-associated herpesvirus latency and negatively regulates K-Rta by antagonizing the RBP-Jkappa coactivator. J. Virol..

[81] Wang S.E., Wu F.Y., Chen H., Shamay M., Zheng Q., Hayward G.S. (2004). Early activation of the Kaposi’s sarcoma-associated herpesvirus RTA, RAP, and MTA promoters by the tetradecanoyl phorbol acetate-induced AP1 pathway. J. Virol..

[82] He Z., Liu Y., Liang D., Wang Z., Robertson E.S., Lan K. (2010). Cellular corepressor TLE2 inhibits replication-and-transcription- activator-mediated transactivation and lytic reactivation of Kaposi’s sarcoma-associated herpesvirus. J. Virol..

[83] Chang P.J., Miller G. (2004). Autoregulation of DNA binding and protein stability of Kaposi’s sarcoma-associated herpesvirus ORF50 protein. J. Virol..

[84] Chang P.J., Shedd D., Miller G. (2008). A mobile functional region of Kaposi’s sarcoma-associated herpesvirus ORF50 protein independently regulates DNA binding and protein abundance. J. Virol..

[85] Peng L., Wu T.T., Tchieu J.H., Feng J., Brown H.J., Feng J., Li X., Qi J., Deng H., Vivanco I., Mellinghoff I.K., Jamieson C., Sun R. (2010). Inhibition of the phosphatidylinositol 3-kinase-Akt pathway enhances gamma-2 herpesvirus lytic replication and facilitates reactivation from latency. J. Gen. Virol..

[86] Cheng F., Weidner-Glunde M., Varjosalo M., Rainio E.M., Lehtonen A., Schulz T.F., Koskinen P.J., Taipale J., Ojala P.M. (2009). KSHV reactivation from latency requires Pim-1 and Pim-3 kinases to inactivate the latency-associated nuclear antigen LANA. PLoS Pathog..

[87] Sandford G., Choi Y.B., Nicholas J. (2009). Role of ORF74-encoded viral G protein-coupled receptor in human herpesvirus 8 lytic replication. J. Virol..

[88] Bottero V., Sharma-Walia N., Kerur N., Paul A.G., Sadagopan S., Cannon M., Chandran B. (2009). Kaposi sarcoma-associated herpes virus (KSHV) G protein-coupled receptor (vGPCR) activates the ORF50 lytic switch promoter: A potential positive feedback loop for sustained ORF50 gene expression. Virology.

[89] Davis D.A., Rinderknecht A.S., Zoeteweij J.P., Aoki Y., Read-Connole E.L., Tosato G., Blauvelt A., Yarchoan R. (2001). Hypoxia induces lytic replication of Kaposi sarcoma-associated herpesvirus. Blood.

[90] Haque M., Davis D.A., Wang V., Widmer I., Yarchoan R. (2003). Kaposi’s sarcoma-associated herpesvirus (human herpesvirus 8) contains hypoxia response elements: relevance to lytic induction by hypoxia. J. Virol..

[91] Wilson S.J., Tsao E.H., Webb B.L., Ye H., Dalton-Griffin L., Tsantoulas C., Gale C.V., Du M.Q., Whitehouse A., Kellam P. (2007). X box binding protein XBP-1s transactivates the Kaposi’s sarcoma-associated herpesvirus (KSHV) ORF50 promoter, linking plasma cell differentiation to KSHV reactivation from latency. J. Virol..

[92] Dalton-Griffin L., Wilson S.J., Kellam P. (2009). X-box binding protein 1 contributes to induction of the Kaposi’s sarcoma-associated herpesvirus lytic cycle under hypoxic conditions. J. Virol..

[93] Huang Y.T., Lin J.K., Lee M.T. (1999). Inhibition of 12-O-tetradecanoylphorbol-13-acetate induction of c-fos mRNA by the protein kinase A inhibitor N-[2-(p-bromocinnamylamino)ethyl]-5-isoquinoline sulfonamide. Biochem. Pharmacol..

[94] Gwack Y., Hwang S., Lim C., Won Y.S., Lee C.H., Choe J. (2002). Kaposi’s Sarcoma-associated herpesvirus open reading frame 50 stimulates the transcriptional activity of STAT3. J. Biol. Chem..

[95] Spadavecchia S., Palmeri D., Lukac D.M. (2009). KSHV Mta protein stimulates transcriptional elongation.

[96] Conrad N.K. (2009). Posttranscriptional gene regulation in Kaposi’s sarcoma-associated herpesvirus. Adv. Appl. Microbiol..

[97] Artavanis-Tsakonas S., Rand M.D., Lake R.J. (1999). Notch signaling: Cell fate control and signal integration in development. Science.

[98] Liu Z.J., Shirakawa T., Li Y., Soma A., Oka M., Dotto G.P., Fairman R.M., Velazquez O.C., Herlyn M. (2003). Regulation of Notch1 and Dll4 by vascular endothelial growth factor in arterial endothelial cells: Implications for modulating arteriogenesis and angiogenesis. Mol. Cell Biol..

[99] Curry C.L., Reed L.L., Broude E., Golde T.E., Miele L., Foreman K.E. (2007). Notch inhibition in Kaposi’s sarcoma tumor cells leads to mitotic catastrophe through nuclear factor-kappaB signaling. Mol. Cancer Ther..

[100] Liu R., Li X., Tulpule A., Zhou Y., Scehnet J.S., Zhang S., Lee J.S., Chaudhary P.M., Jung J., Gill P.S. (2010). KSHV-induced notch components render endothelial and mural cell characteristics and cell survival. Blood.

[101] Emuss V., Lagos D., Pizzey A., Gratrix F., Henderson S.R., Boshoff C. (2009). KSHV manipulates Notch signaling by DLL4 and JAG1 to alter cell cycle genes in lymphatic endothelia. PloS Pathog..

[102] Curry C.L., Reed L.L., Golde T.E., Miele L., Nickoloff B.J., Foreman K.E. (2005). Gamma secretase inhibitor blocks Notch activation and induces apoptosis in Kaposi’s sarcoma tumor cells. Oncogene.

[103] Lan K., Choudhuri T., Murakami M., Kuppers D.A., Robertson E.S. (2006). Intracellular activated Notch1 is critical for proliferation of Kaposi’s sarcoma-associated herpesvirus-associated B-lymphoma cell lines *in vitro*. J. Virol..

[104] Lan K., Murakami M., Choudhuri T., Kuppers D.A., Robertson E.S. (2006). Intracellular-activated Notch1 can reactivate Kaposi’s sarcoma-associated herpesvirus from latency. Virology.

[105] Gonzalez-Lopez O., Palmeri D., Lukac D.M. (2015). Comprehensive analysis of DNA sequences required for KSHV Rta to stimulate DNA binding of the Notch effector RBP-Jk.

[106] Ellison T.J., Izumiya Y., Izumiya C., Luciw P.A., Kung H.J. (2009). A comprehensive analysis of recruitment and transactivation potential of K-Rta and K-bZIP during reactivation of Kaposi’s sarcoma-associated herpesvirus. Virology.

[107] Spadavecchia S., Gonzalez-Lopez O., Carroll K.D., Palmeri D., Lukac D.M. (2010). Convergence of Kaposi’s sarcoma-associated herpesvirus reactivation with Epstein-Barr virus latency and cellular growth mediated by the notch signaling pathway in coinfected cells. J. Virol..

[108] Schaefer M.H., Wanker E.E., Andrade-Navarro M.A. (2012). Evolution and function of CAG/polyglutamine repeats in protein-protein interaction networks. Nucleic Acids Res..

[109] Williamson M.P. (1994). The structure and function of proline-rich regions in proteins. Biochem. J..

[110] Chang M., Brown H.J., Collado-Hidalgo A., Arevalo J.M., Galic Z., Symensma T.L., Tanaka L., Deng H., Zack J.A., Sun R., Cole S.W. (2005). beta-Adrenoreceptors reactivate Kaposi’s sarcoma-associated herpesvirus lytic replication via PKA-dependent control of viral RTA. J. Virol..

[111] Gothel S.F., Marahiel M.A. (1999). Peptidyl-prolyl cis-trans isomerases, a superfamily of ubiquitous folding catalysts. Cell Mol. Life Sci..

[112] Anfinsen C.B. (1973). Principles that govern the folding of protein chains. Science.

[113] Schmid F.X. (1995). Protein folding. Prolyl isomerases join the fold. Curr. Biol..

[114] Dolinski K., Muir S., Cardenas M., Heitman J. (1997). All cyclophilins and FK506 binding proteins are, individually and collectively, dispensable for viability in Saccharomyces cerevisiae. Proc. Natl. Acad. Sci. USA.

[115] Lu K.P., Finn G., Lee T.H., Nicholson L.K. (2007). Prolyl cis-trans isomerization as a molecular timer. Nat. Chem. Biol..

[116] Scholz C., Rahfeld J., Fischer G., Schmid F.X. (1997). Catalysis of protein folding by parvulin. J. Mol. Biol..

[117] Pavlov M.Y., Watts R.E., Tan Z., Cornish V.W., Ehrenberg M., Forster A.C. (2009). Slow peptide bond formation by proline and other N-alkylamino acids in translation. Proc. Natl. Acad. Sci. USA.

[118] Lee S., Tsai F.T. (2005). Molecular chaperones in protein quality control. J. Biochem. Mol. Biol..

[119] Lu K.P. (2004). Pinning down cell signaling, cancer and Alzheimer's disease. Trends Biochem. Sci..

[120] Frausto S.D., Lee E., Tang H. (2013). Cyclophilins as modulators of viral replication. Viruses.

[121] Lu K.P., Hanes S.D., Hunter T. (1996). A human peptidyl-prolyl isomerase essential for regulation of mitosis. Nature.

[122] O'Connell M.J., Krien M.J., Hunter T. (2003). Never say never. The NIMA-related protein kinases in mitotic control. Trends Cell Biol..

[123] Ranganathan R., Lu K.P., Hunter T., Noel J.P. (1997). Structural and functional analysis of the mitotic rotamase Pin1 suggests substrate recognition is phosphorylation dependent. Cell.

[124] Shen M., Haggblom C., Vogt M., Hunter T., Lu K.P. (1997). Characterization and cell cycle regulation of the related human telomeric proteins Pin2 and TRF1 suggest a role in mitosis. Proc. Natl. Acad. Sci. USA.

[125] Lee T.H., Perrem K., Harper J.W., Lu K.P., Zhou X.Z. (2006). The F-box protein FBX4 targets PIN2/TRF1 for ubiquitin-mediated degradation and regulates telomere maintenance. J. Biol. Chem..

[126] Takahashi K., Uchida C., Shin R.W., Shimazaki K., Uchida T. (2008). Prolyl isomerase, Pin1: New findings of post-translational modifications and physiological substrates in cancer, asthma and Alzheimer’s disease. Cell Mol Life Sci..

[127] Lu K.P., Zhou X.Z. (2007). The prolyl isomerase PIN1: A pivotal new twist in phosphorylation signalling and disease. Nat. Rev. Mol. Cell Biol..

[128] Hanes S.D. (2014). The Ess1 prolyl isomerase: Traffic cop of the RNA polymerase II transcription cycle. Biochim. Biophys. Acta.

[129] Scholz C., Scherer G., Mayr L.M., Schindler T., Fischer G., Schmid F.X. (1998). Prolyl isomerases do not catalyze isomerization of non-prolyl peptide bonds. Biol. Chem..

[130] Lu K.P., Liou Y.C., Zhou X.Z. (2002). Pinning down proline-directed phosphorylation signaling. Trends Cell Biol..

[131] Kojima Y., Ryo A. (2010). Pinning down viral proteins: A new prototype for virus-host cell interaction. Front. Microbiol..

[132] Wulf G., Finn G., Suizu F., Lu K.P. (2005). Phosphorylation-specific prolyl isomerization: is there an underlying theme?. Nat. Cell Biol..

[133] Liou Y.C., Zhou X.Z., Lu K.P. (2011). Prolyl isomerase Pin1 as a molecular switch to determine the fate of phosphoproteins. Trends Biochem. Sci..

[134] Wulf G.M., Ryo A., Wulf G.G., Lee S.W., Niu T., Petkova V., Lu K.P. (2001). Pin1 is overexpressed in breast cancer and cooperates with Ras signaling in increasing the transcriptional activity of c-Jun towards cyclin D1. EMBO J..

[135] Bao L., Kimzey A., Sauter G., Sowadski J.M., Lu K.P., Wang D.G. (2004). Prevalent overexpression of prolyl isomerase Pin1 in human cancers. Am. J. Pathol..

[136] Liou Y.C., Ryo A., Huang H.K., Lu P.J., Bronson R., Fujimori F., Uchida T., Hunter T., Lu K.P. (2002). Loss of Pin1 function in the mouse causes phenotypes resembling cyclin D1-null phenotypes. Proc. Natl. Acad. Sci. USA.

[137] Xu Y.X., Hirose Y., Zhou X.Z., Lu K.P., Manley J.L. (2003). Pin1 modulates the structure and function of human RNA polymerase II. Genes Dev..

[138] Wildemann D., Hernandez Alvarez B., Stoller G., Zhou X.Z., Lu K.P., Erdmann F., Ferrari D., Fischer G. (2007). An essential role for Pin1 in Xenopus laevis embryonic development revealed by specific inhibitors. Biol. Chem..

[139] Lee T.H., Tun-Kyi A., Shi R., Lim J., Soohoo C., Finn G., Balastik M., Pastorino L., Wulf G., Zhou X.Z., Lu K.P. (2009). Essential role of Pin1 in the regulation of TRF1 stability and telomere maintenance. Nat. Cell Biol..

[140] Raghuram N., Strickfaden H., McDonald D., Williams K., Fang H., Mizzen C., Hayes J.J., Th'ng J., Hendzel M.J. (2013). Pin1 promotes histone H1 dephosphorylation and stabilizes its binding to chromatin. J. Cell Biol..

[141] Krishnan N., Titus M.A., Thapar R. (2014). The prolyl isomerase pin1 regulates mRNA levels of genes with short half-lives by targeting specific RNA binding proteins. PLoS One.

[142] Wulf G., Garg P., Liou Y.C., Iglehart D., Lu K.P. (2004). Modeling breast cancer *in vivo* and *ex vivo* reveals an essential role of Pin1 in tumorigenesis. EMBO J..

[143] Kim C.J., Cho Y.G., Park Y.G., Nam S.W., Kim S.Y., Lee S.H., Yoo N.J., Lee J.Y., Park W.S. (2005). Pin1 overexpression in colorectal cancer and its correlation with aberrant beta-catenin expression. World J. Gastroenterol..

[144] Ryo A., Uemura H., Ishiguro H., Saitoh T., Yamaguchi A., Perrem K., Kubota Y., Lu K.P., Aoki I. (2005). Stable suppression of tumorigenicity by Pin1-targeted RNA interference in prostate cancer. Clin. Cancer Res..

[145] Wang H., Zhang J., Feng W., Zhang S., Liang H., Wang Y., Zheng Q., Li Z. (2007). PIN1 gene overexpression and beta-catenin gene mutation/expression in hepatocellular carcinoma and their significance. J. Huazhong Univ. Sci. Technolog. Med. Sci..

[146] Kim M.R., Choi H.S., Heo T.H., Hwang S.W., Kang K.W. (2008). Induction of vascular endothelial growth factor by peptidyl-prolyl isomerase Pin1 in breast cancer cells. Biochem. Biophys. Res. Commun..

[147] Rustighi A., Tiberi L., Soldano A., Napoli M., Nuciforo P., Rosato A., Kaplan F., Capobianco A., Pece S., Di Fiore P.P., Del Sal G. (2009). The prolyl-isomerase Pin1 is a Notch1 target that enhances Notch1 activation in cancer. Nat. Cell Biol..

[148] Khanal P., Kim G., Yun H.J., Cho H.G., Choi H.S. (2013). The prolyl isomerase Pin1 interacts with and downregulates the activity of AMPK leading to induction of tumorigenicity of hepatocarcinoma cells. Mol. Carcinog..

[149] Suizu F., Ryo A., Wulf G., Lim J., Lu K.P. (2006). Pin1 regulates centrosome duplication, and its overexpression induces centrosome amplification, chromosome instability, and oncogenesis. Mol. Cell Biol..

[150] Atkinson G.P., Nozell S.E., Harrison D.K., Stonecypher M.S., Chen D., Benveniste E.N. (2009). The prolyl isomerase Pin1 regulates the NF-kappaB signaling pathway and interleukin-8 expression in glioblastoma. Oncogene.

[151] Lee N.Y., Choi H.K., Shim J.H., Kang K.W., Dong Z., Choi H.S. (2009). The prolyl isomerase Pin1 interacts with a ribosomal protein S6 kinase to enhance insulin-induced AP-1 activity and cellular transformation. Carcinogenesis.

[152] Pulikkan J.A., Dengler V., Peer Zada A.A., Kawasaki A., Geletu M., Pasalic Z., Bohlander S.K., Ryo A., Tenen D.G., Behre G. (2010). Elevated PIN1 expression by C/EBPalpha-p30 blocks C/EBPalpha-induced granulocytic differentiation through c-Jun in AML. Leukemia.

[153] Ayala G., Wang D., Wulf G., Frolov A., Li R., Sowadski J., Wheeler T.M., Lu K.P., Bao L. (2003). The prolyl isomerase Pin1 is a novel prognostic marker in human prostate cancer. Cancer Res..

[154] Dochi T., Nakano T., Inoue M., Takamune N., Shoji S., Sano K., Misumi S. (2014). Phosphorylation of human immunodeficiency virus type 1 (HIV-1) capsid protein at serine 16, required for peptidyl-prolyl isomerase (Pin1)-dependent uncoating, is mediated by virion-incorporated extracellular signal-regulated kinase 2 (ERK2). J. Gen. Virol..

[155] Misumi S., Inoue M., Dochi T., Kishimoto N., Hasegawa N., Takamune N., Shoji S. (2010). Uncoating of human immunodeficiency virus type 1 requires prolyl isomerase Pin1. J. Biol. Chem..

[156] Watashi K., Khan M., Yedavalli V.R., Yeung M.L., Strebel K., Jeang K.T. (2008). Human immunodeficiency virus type 1 replication and regulation of APOBEC3G by peptidyl prolyl isomerase Pin1. J. Virol..

[157] Saitoh T., Tun-Kyi A., Ryo A., Yamamoto M., Finn G., Fujita T., Akira S., Yamamoto N., Lu K.P., Yamaoka S. (2006). Negative regulation of interferon-regulatory factor 3-dependent innate antiviral response by the prolyl isomerase Pin1. Nat. Immunol..

[158] Lim Y.S., Tran H.T., Park S.J., Yim S.A., Hwang S.B. (2011). Peptidyl-prolyl isomerase Pin1 is a cellular factor required for hepatitis C virus propagation. J. Virol..

[159] Avila M.A., Lu K.P. (2007). Hepatitis B virus x protein and pin1 in liver cancer: “les liaisons dangereuses”. Gastroenterology.

[160] Chowdhury I.H., Radonovich M., Mahieux R., Pise-Masison C., Muralidhar S., Brady J.N. (2003). P53 facilitates degradation of human T-cell leukaemia virus type I Tax-binding protein through a proteasome-dependent pathway. J. Gen. Virol..

[161] Peloponese J.M., Yasunaga J., Kinjo T., Watashi K., Jeang K.T. (2009). Peptidylproline cis-trans-isomerase Pin1 interacts with human T-cell leukemia virus type 1 tax and modulates its activation of NF-kappaB. J. Virol..

[162] Milbradt J., Webel R., Auerochs S., Sticht H., Marschall M. (2010). Novel mode of phosphorylation-triggered reorganization of the nuclear lamina during nuclear egress of human cytomegalovirus. J. Biol. Chem..

[163] Ott D.E., Coren L.V., Johnson D.G., Kane B.P., Sowder R.C., Kim Y.D., Fisher R.J., Zhou X.Z., Lu K.P., Henderson L.E. (2000). Actin-binding cellular proteins inside human immunodeficiency virus type 1. Virology.

[164] Kamimoto T., Zama T., Aoki R., Muro Y., Hagiwara M. (2001). Identification of a novel kinesin-related protein, KRMP1, as a target for mitotic peptidyl-prolyl isomerase Pin1. J. Biol. Chem..

[165] Narita Y., Murata T., Ryo A., Kawashima D., Sugimoto A., Kanda T., Kimura H., Tsurumi T. (2013). Pin1 interacts with the Epstein-Barr virus DNA polymerase catalytic subunit and regulates viral DNA replication. J. Virol..

[166] Guito J., Gavina A., Palmeri D., Lukac D.M. (2014). The cellular peptidyl-prolyl *cis/trans* isomerase Pin1 regulates reactivation of Kaposi’s sarcoma-associated herpesvirus from latency. J. Virol..

[167] Gantt S., Carlsson J., Ikoma M., Gachelet E., Gray M., Geballe A.P., Corey L., Casper C., Lagunoff M., Vieira J. (2011). The HIV protease inhibitor nelfinavir inhibits Kaposi’s sarcoma-associated herpesvirus replication *in vitro*. Antimicrob. Agents Chemother..

[168] Toth Z., Brulois K.F., Wong L.Y., Lee H.R., Chung B., Jung J.U. (2012). Negative elongation factor-mediated suppression of RNA polymerase II elongation of Kaposi’s sarcoma-associated herpesvirus lytic gene expression. J. Virol..

[169] Feldman D.E., Chauhan V., Koong A.C. (2005). The unfolded protein response: A novel component of the hypoxic stress response in tumors. Mol. Cancer Res..

[170] Koumenis C., Bi M., Ye J., Feldman D., Koong A.C. (2007). Hypoxia and the unfolded protein response. Methods Enzymol..

[171] Cai Q.L., Knight J.S., Verma S.C., Zald P., Robertson E.S. (2006). EC5S ubiquitin complex is recruited by KSHV latent antigen LANA for degradation of the VHL and p53 tumor suppressors. PLoS Pathog..

[172] Long E., Ilie M., Hofman V., Havet K., Selva E., Butori C., Lacour J.P., Nelson A.M., Cathomas G., Hofman P. (2009). LANA-1, Bcl-2, Mcl-1 and HIF-1alpha protein expression in HIV-associated Kaposi sarcoma. Virchows Arch..

[173] Kim M.R., Choi H.S., Yang J.W., Park B.C., Kim J.A., Kang K.W. (2009). Enhancement of vascular endothelial growth factor-mediated angiogenesis in tamoxifen-resistant breast cancer cells: Role of Pin1 overexpression. Mol. Cancer Ther..

[174] Ryo A., Nakamura M., Wulf G., Liou Y.C., Lu K.P. (2001). Pin1 regulates turnover and subcellular localization of beta-catenin by inhibiting its interaction with APC. Nat. Cell Biol..

[175] Cho Y.S., Park S.Y., Kim D.J., Lee S.H., Woo K.M., Lee K.A., Lee Y.J., Cho Y.Y., Shim J.H. (2012). TPA-induced cell transformation provokes a complex formation between Pin1 and 90 kDa ribosomal protein S6 kinase 2. Mol. Cell Biochem..

[176] Xu Y.X., Manley J.L. (2004). Pinning down transcription: Regulation of RNA polymerase II activity during the cell cycle. Cell Cycle.

[177] Xu Y.X., Manley J.L. (2007). Pin1 modulates RNA polymerase II activity during the transcription cycle. Genes Dev..

[178] Wulf G.M., Liou Y.C., Ryo A., Lee S.W., Lu K.P. (2002). Role of Pin1 in the regulation of p53 stability and p21 transactivation, and cell cycle checkpoints in response to DNA damage. J. Biol. Chem..

[179] Zheng H., You H., Zhou X.Z., Murray S.A., Uchida T., Wulf G., Gu L., Tang X., Lu K.P., Xiao Z.X. (2002). The prolyl isomerase Pin1 is a regulator of p53 in genotoxic response. Nature.

[180] Liao Y., Wei Y., Zhou X., Yang J.Y., Dai C., Chen Y.J., Agarwal N.K., Sarbassov D., Shi D., Yu D., Hung M.C. (2009). Peptidyl-prolyl cis/trans isomerase Pin1 is critical for the regulation of PKB/Akt stability and activation phosphorylation. Oncogene.

[181] Lu P.J., Zhou X.Z., Liou Y.C., Noel J.P., Lu K.P. (2002). Critical role of WW domain phosphorylation in regulating phosphoserine binding activity and Pin1 function. J. Biol. Chem..

[182] Lavoie S.B., Albert A.L., Handa H., Vincent M., Bensaude O. (2001). The peptidyl-prolyl isomerase Pin1 interacts with hSpt5 phosphorylated by Cdk9. J. Mol. Biol..

[183] Lan K., Verma S.C., Murakami M., Bajaj B., Kaul R., Robertson E.S. (2007). Kaposi’s sarcoma herpesvirus-encoded latency-associated nuclear antigen stabilizes intracellular activated Notch by targeting the Sel10 protein. Proc. Natl. Acad. Sci. USA.

[184] Kirshner J.R., Lukac D.M., Chang J., Ganem D. (2000). Kaposi’s sarcoma-associated herpesvirus open reading frame 57 encodes a posttranscriptional regulator with multiple distinct activities. J. Virol..

